# VNIR Hyperspectral Signatures for Early Detection and Machine-Learning Classification of Wheat Diseases

**DOI:** 10.3390/plants14233644

**Published:** 2025-11-29

**Authors:** Rimma M. Ualiyeva, Mariya M. Kaverina, Anastasiya V. Osipova, Yernar B. Kairbayev, Sayan B. Zhangazin, Nurgul N. Iksat, Nariman B. Mapitov

**Affiliations:** 1 Department of Biology and Ecology, Toraighyrov University, Pavlodar 140008, Kazakhstan; ualiyeva.r@gmail.com (R.M.U.); aanastasiyaaa@internet.ru (A.V.O.); ernarkairbaev@gmail.com (Y.B.K.); mapitov@mail.ru (N.B.M.); 2 Department of Biotechnology and Microbiology, L.N. Gumilyov Eurasian National University, Astana 010000, Kazakhstan; sayanzhangazin@gmail.com (S.B.Z.); nurguliksat@gmail.com (N.N.I.)

**Keywords:** hyperspectral imaging, plant proximal hyperspectral sensing, spring wheat, phytopathogens, spectral signatures, machine learning, automated disease classification, phytosanitary monitoring

## Abstract

This article presents the results of a comprehensive study aimed at developing automated diagnostic methods for identifying spring wheat phytopathologies using hyperspectral imaging (HSI). The research aimed to create an effective plant disease detection system, including at the early stages, which is critically important for ensuring food security in regions where wheat plays a key role in the agro-industrial sector. The study analyses the spectral characteristics of major wheat diseases, including powdery mildew, fusarium head blight, septoria glume blotch, root rots, various types of leaf spots, brown rust, and loose smut. Healthy plants differ from diseased ones in that they show a mostly uniform tone without distinct spots or patches on hyperspectral images, and their spectra have a consistent shape without sharp fluctuations. In contrast, disease spectra, differ sharply from those of healthy areas and can take diverse forms. Wheat diseases with a light coating (powdery mildew, fusarium head blight) exhibit high reflectance; chlorosis in the early stages of diseases (rust, leaf spot, septoria leaf blotch) exhibits curves with medium reflectance, and diseases with dark colouration (loose smut, root rot) have low reflectance values. These differences in reflectance among fungal diseases are caused by pigments produced by the pathogens, which either strongly absorb light or reflect most of it. The presence or absence of pigment production is determined by adaptive mechanisms. Based on these patterns in the spectral characteristics and optical properties of the diseases, a classification model was developed with 94% overall accuracy. Random Forest proved to be the most effective method for the automated detection of wheat phytopathogens using hyperspectral data. The practical significance of this research lies in the potential integration of the developed phytopathology detection approach into precision agriculture systems and the use of UAV platforms, enabling rapid large-scale crop monitoring for the timely detection. The study’s results confirm the promising potential of combining hyperspectral technologies and machine learning methods for monitoring the phytosanitary condition of crops. Our findings contribute to the advancement of digital agriculture and are particularly valuable for the agro-industrial sector of Central Asia, where adopting precision farming technologies is a strategic priority given the climatic risks and export-oriented nature of grain production.

## Simple Summary

This study explores the use of hyperspectral imaging to detect spring wheat diseases at various stages of their development, based on the distinctive spectral characteristics of healthy and diseased plants. The research focuses on several common wheat diseases, including powdery mildew, fusarium head blight, root rot, various leaf spot diseases, septoria leaf spot, brown rust, and loose smut. It was found that diseases with a light coating reflect more light and show high reflectance values (60–80%), whereas diseases accompanied by dark spores absorb most of the incoming radiation and exhibit low reflectance values (7–10%). These differences create distinct spectral patterns that allow reliable differentiation between types of infections. Using these patterns, a machine learning classification model based on the Random Forest algorithm was developed to detect wheat diseases automatically with a high accuracy of 94%. This method outperforms other machine learning approaches in both qualitative and quantitative metrics of disease detection. The study demonstrates that combining hyperspectral imaging with computer vision and machine learning provides an effective tool for monitoring plant health. This approach is especially valuable in regions where wheat is a critical component of food security. Accurate disease detection enables farmers to take timely, targeted action, reducing crop losses and minimising pesticide use—thereby promoting more sustainable agricultural practices.

## 1. Introduction

Hyperspectral imaging (HSI) is a rapidly advancing remote sensing technology with growing adoption in agriculture. The technology captures reflected and emitted light across hundreds of narrow spectral bands, enabling the detection of physiological and biochemical characteristics of crops at early stages. In recent years, interest in HSI has grown significantly [1,2].

In agriculture, this technology is applied for a variety of purposes. Currently, hyperspectral methods are used to obtain information about the biophysical properties of plants based on pigment content, water, nitrogen, protein, structural elements, and leaf area index (LAI) [3,4,5]. Hyperspectral imaging, combined with deep learning, has proven highly accurate in segmenting plants and detecting physiological changes in oat genotypes exposed to salinity stress. This approach speeds up the selection of varieties that are more tolerant to stress [6,7]. In practical agricultural settings, hyperspectral technology is implemented through various platforms: laboratory scanners (for seed and grain analysis), unmanned aerial vehicles (UAVs), manned aircraft, and satellite systems [8,9,10,11]. UAV platforms provide high spatial resolution and enable rapid monitoring of individual fields, while satellite systems cover large areas with high revisit frequency. Hyperspectral data acquired by UAVs enable precise prediction of winter wheat traits such as biomass, height, LAI, and canopy cover (CC), with Partial Least Squares Regression (PLSR) and Random Forest (RF) models significantly outperforming simple vegetation indices [12,13,14,15]. Moreover, this approach enables accurate yield prediction and the measurement of key quality traits of durum wheat (protein content, vitreousness, test weight) well before harvest [16,17], as well as the characterisation of metabolite profiles in different organs of durum wheat [12]. Using UAV-based hyperspectral data and hybrid SVM-XGBoost models, wheat varieties can be accurately distinguished under varying levels of nitrogen stress, supporting optimised fertilisation strategies [15,18,19]. Combining UAV-based HSI with machine learning enables precise detection and quantification of bacterial leaf streak in wheat, speeding up the selection of resistant varieties and large-scale disease monitoring [20,21,22,23]. Integrating hyperspectral and thermal imaging on UAV platforms enables accurate assessment of wheat physiological traits (e.g., stomatal conductance) and identification of associated genetic markers, thus accelerating the breeding of climate-resilient cultivars [24,25,26,27]. In laboratory conditions, HSI is employed for precise grain quality assessment and quality control at grain elevators [8,28,29,30,31].

The choice of spectral range is of particular importance. The visible range (VIS) corresponds to pigments, near-infrared (NIR) relates to water content, and short-wave infrared (SWIR) corresponds to proteins and tissue structure [4,32]. To improve accuracy, new spectral indices and feature selection algorithms are actively being developed alongside machine learning and deep learning methods. Studies show that VIS, NIR, and SWIR bands carry different biophysical markers such as chlorophyll, water, and protein [33,34,35]. HSI allows the detection of wheat stress caused by drought, nitrogen deficiency, diseases, or pest infestations at earlier stages than visual inspections or multispectral imaging [36]. Machine learning methods (e.g., Random Forest, SVM) and neural networks achieve high classification accuracy for stress types, ranging from 80% to 95% [37,38,39,40].

Major wheat diseases differ significantly in their mechanisms of infection and the physiological changes they induce. Brown leaf rust (*Puccinia triticina*) causes the formation of uredinial pustules and leads to reduced chlorophyll content, suppression of photosynthesis, and accelerated tissue senescence [41,42]. Septoria leaf blotch (*Zymoseptoria tritici*) is characterised by necrotic lesions with pycnidia and is accompanied by the destruction of mesophyll structure, which substantially affects the reflectance properties of the leaf surface [43,44]. Powdery mildew (*Blumeria graminis*) forms a superficial mycelial coating, disrupts gas exchange, and reduces the intensity of photosynthetic processes [45]. Root and crown rots, primarily of fusarium origin, cause impaired water conductivity and suppressed growth processes, which manifest as spectral changes in the visible and near-infrared ranges. These widespread diseases are major contributors to yield losses globally, highlighting the need for advanced tools for their early detection. However, most HSI studies focus on single pathogens specific infection stages, or particular regions, which limits model generalisability and creates a gap in comprehensive local spectral libraries.

Hyperspectral data demonstrate competitive performance for wheat yield estimation compared to multispectral data. Hyperspectral imaging (HSI) proves particularly effective during the grain-filling and ripening stages, when spectral characteristics are closely linked to biochemical indicators of yield [46]. The most informative period for remote identification is from 45 to 80 days after sowing [47]. A VNIR hyperspectral system (400–1000 nm) has demonstrated high accuracy in distinguishing between healthy, sprouted, and severely sprouted wheat grains based on spectral and morphological features [48,49]. When combined with PCA and LDA, it achieved up to 92% accuracy in detecting and classifying the severity of Fusarium infection in wheat [50]. Integrated with machine learning, HSI can detect septoria disease up to 7 days before visual symptoms appear, with up to 97% accuracy and reliable disease progression prediction [51]. Hyperspectral profiling has also identified distinct changes in the spectral signatures of wheat spikes and flag leaves immediately following frost events, confirming the feasibility of remote frost damage monitoring [52]. It has been effectively used to estimate yield and nitrogen uptake in spring wheat with high accuracy, although predictions of grain nitrogen content remain limited and require further development for fertiliser applications [53]. Reflectance typically increases during the vegetative stage and declines by maturation, which is associated with reduced plant moisture and changes in leaf structure [54]. Using fractional-order spectral transformations and an optimised SMA-LSSSVM model, HSI enabled precise, non-destructive chlorophyll content (SPAD) estimation in spring wheat at the stem elongation stage [55]. In addition, an ANN-based model was successfully applied to estimate chlorophyll content in wheat leaves [56]. Moreover, optimised multiband spectral indices and machine learning algorithms (KNN, SVM, ANN) allowed accurate estimation of leaf water content in spring wheat [57]. Hyperspectral data combined with multivariate modelling techniques (PLS, LASSO, E.net) has shown high accuracy in predicting physiological and yield traits under varying water regimes, supporting faster breeding cycles and improving drought resilience in crops [58]. Integrating canopy-level data with environmental variables like soil temperature and moisture, along with machine learning, significantly enhances the prediction accuracy of the water status in winter wheat [59]. In laboratory settings, HSI is widely used for assessing grain moisture and protein content, as well as detecting impurities and mycotoxins. These approaches enable fast and automated quality control, which is particularly important for export-orientated grain production [60].

Overall, hyperspectral imaging is a promising tool for assessing wheat quality, enabling the detection of composition, damage, and adulteration. Recent studies affirm the effectiveness of this technology and highlight its strong potential for contributing to a more sustainable food supply chain [61].

A critical review of the literature reveals a common limitation: many successful models for disease detection are developed under controlled conditions or for specific geographic regions, and their performance is highly dependent on the representativeness of the spectral library used for training [1,62]. Furthermore, a lack of systematic comparative studies benchmarking a wide range of classifiers—from simpler linear models to complex ensemble methods—on the same dataset of multiple wheat diseases, makes it difficult to identify the most robust approach [63]. This gap is particularly relevant for regions with unique agro-climatic conditions, where spectral signatures of diseases may differ from established models [64]. Most existing spectral libraries focus on post-harvest grain analysis rather than in-field disease detection [65,66].

Recent years have seen a sharp increase in research integrating hyperspectral data with machine and deep learning. Hyperspectral imaging has become a powerful tool for promoting sustainable and efficient agricultural production. In Central Asia, its relevance is especially high due to climate risks, the need for efficient water use, and the goal of improving grain yield and quality. As one of the world’s leading wheat producers, Kazakhstan ranks among the top ten grain exporters, shipping wheat products to over 70 countries [67]. Wheat also plays a crucial role in ensuring regional food security. Therefore, adopting advanced technologies for precision monitoring and early disease detection is particularly important.

Despite the growing global body of research on hyperspectral technologies in wheat studies, their application in Central Asian countries remains limited and insufficiently implemented in practice. This highlights a significant scientific gap: the absence of a localised spectral library and validated disease detection methodology tailored to the spring wheat systems of this crucial grain-producing region. We hypothesise that hyperspectral imaging, combined with machine learning models, can accurately detect phytopathologies in spring wheat under local agro-climatic conditions.

This study aims to evaluate the potential of hyperspectral imaging for identifying and detecting early signs of phytopathologies in spring wheat through the analysis of its spectral profiles. The novelty of this study lies in developing a local spectral library of major spring wheat diseases, constructed based on hyperspectral data from naturally infected plants collected in the northeastern region of Kazakhstan. For the first time, a comprehensive analysis of the spectral characteristics of infected leaves was conducted using VNIR hyperspectral imaging (400–1000 nm, 1200 spectral channels), followed by disease classification using machine learning methods. Additionally, a comparative study of the performance of various algorithms (PLS-DA, SVM, Random Forest, SIMCA, etc.) was carried out, which allowed for the identification of the most effective approaches for automated phytopathology diagnosis. The obtained results demonstrate the potential of hyperspectral imaging for early disease detection, even before the appearance of pronounced visual symptoms.

## 2. Materials and Methods

### 2.1. Objects and Sampling Areas for Spectral Analysis

The study focused on spring wheat (*Triticum aestivum* L., 1753) samples infected with the most common fungal pathogens of cereal crops, including *Fusarium* spp., *Bipolaris sorokiniana* (Sacc., 1890), *Zymoseptoria tritici* (syn. *Septoria tritici*, Desm., 1842), *Pyrenophora tritici-repentis* (Died., 1923) Drechsler, *Cochliobolus sativus* (S. Ito & Kurib., 1942), *Puccinia graminis* (Pers., 1797), *Puccinia triticina* (Dietel & Holw., 1899), *Fusarium graminearum* (Schwabe, 1839), *Parastagonospora nodorum* (syn. *Septoria nodorum*, Berk., 1845), *Ustilago tritici* (Pers., 1775) and *Blumeria graminis* f. sp. *tritici* Marchal (Bgt, 1902).

Sampling was carried out in 2025 across the major grain-producing regions of northeastern Kazakhstan, where these phytopathogens are prevalent and cause significant annual yield losses. Both healthy and diseased plants were collected during key phenological growth stages (BBCH (Biologische Bundesanstalt, Bundessortenamt und Chemische Industrie) 25–35, 39–59, 61–75), ensuring that different stages of infection were represented and that a comprehensive spectral dataset was obtained for subsequent analysis and machine learning model development. BBCH scale is used to describe the phenological growth stages of wheat, ensuring accurate characterisation of plant development during hyperspectral data collection.

### 2.2. Assessment of Spring Wheat Infection by Airborne and Foliar-Stem Pathogens

Plant sampling was conducted in the main wheat-growing areas of the Pavlodar region during key phenological phases (BBCH 25–35, 39–59, 61–75). The goal was to create a representative dataset of both healthy and infected plants for spectral analysis and the development of AI-based diagnostic models.

In total, 480 spring wheat plants were surveyed across five field plots with natural infection. Within each plot, samples were collected from 10 survey points, each containing 10 plants, with a spacing of 25–50 steps between plants. Sampling progressed 25–50 m inward from the field edge and extended 200–300 m into the crop stand. For uniformly distributed infections, triangular or rectangular sampling schemes were used, whereas chessboard or diagonal patterns were applied in cases of localised (patchy) disease occurrence [68].

A representative subset of 279 plants was selected for hyperspectral imaging, covering the major disease groups: Fusarium head blight (*n*_1_ = 25), Brown ear and stem rust (*n*_2_ = 23), Septoria glume blotch (*n*_3_ = 18), Fusarium root rot (*n*_4_ = 17), Helminthosporium root rot (*n*_5_ = 31), Septoria tritici blotch (*n*_6_ = 28), Tan spot (*n*_7_ = 25), Spot blotch (*n*_8_ = 26), Leaf rust (*n*_9_ = 31), Loose smut (*n*_10_ = 16), Powdery mildew (*n*_11_ = 9), as well as healthy control plants (*n*_12_ = 30).

The infection severity of each plant was assessed using the CIMMYT visual scale (0–100%), ensuring data comparability and confirming variability in disease intensity.

Disease severity was evaluated according to the CIMMYT visual scale (0–100%), which quantifies the percentage of affected leaf area. A score of 0% indicates the absence of symptoms, 1–20% reflects mild infection, 21–50% indicates moderate infection, and 51–100% corresponds to severe disease development. This scale is widely used for disease phenotyping in cereal crops and enables consistent assessment of infection intensity under field conditions [69].

Infected samples were selected based on characteristic visual symptoms of major wheat diseases, including septoria leaf blotch (*Z. tritici, P. nodorum*), tan spot (*P. tritici-repentis*), rust diseases (*P. triticina, P. graminis*), fusarium head blight (*F. graminearum*), spot blotch (*C. sativus*), loose smut (*U. tritici*), powdery mildew (*B. graminis*), and root rot (*Fusarium* spp., *B. sorokiniana*). Control samples consisted of healthy plants with no visible disease symptoms. Pathogen diagnoses were confirmed using laboratory methods—microscopy and pure culture isolation—according to the State Standard 12044-93 “Methods for determining disease infection” [70].

The collected samples were subsequently used for hyperspectral scanning and for building a training dataset for AI model development and testing.

### 2.3. Hyperspectral Imaging

Laboratory studies were conducted at the Biological Research Laboratory of Toraighyrov University (Pavlodar, Kazakhstan).

#### 2.3.1. Sample Preparation for Hyperspectral Imaging

Plant samples infected with various pathogens were prepared following a standardised procedure. Panels with a flat matte surface were used for calibration, and samples were placed 3–5 cm apart to avoid overlap. Samples were fixed in their natural orientation with both leaf surfaces exposed. White and dark calibration was performed using a Spectralon panel to ensure radiometric accuracy [71].

#### 2.3.2. Hyperspectral Image Acquisition and Processing

##### Technical Parameters and Imaging Mode

Hyperspectral imaging was performed using a FigSpec^®^ FS-13 line-scanning hyperspectral camera (CHNSpec Technology, Hangzhou, China) with a transmission diffraction grating. The system recorded up to 1200 spectral channels (400–1000 nm) with 2.5 nm spectral resolution (FWHM). Spatial resolution was 1920 pixels per line (pixel size 5.86 µm), with imaging at 128 fps (up to 3300 Hz using ROI selection). The CMOS detector provided high sensitivity and low noise. The camera outputs data over a USB 3.0 interface and uses a C-mount lens attachment [35,72].

##### Image Processing and Preparation for Classification

**Figure 1:**
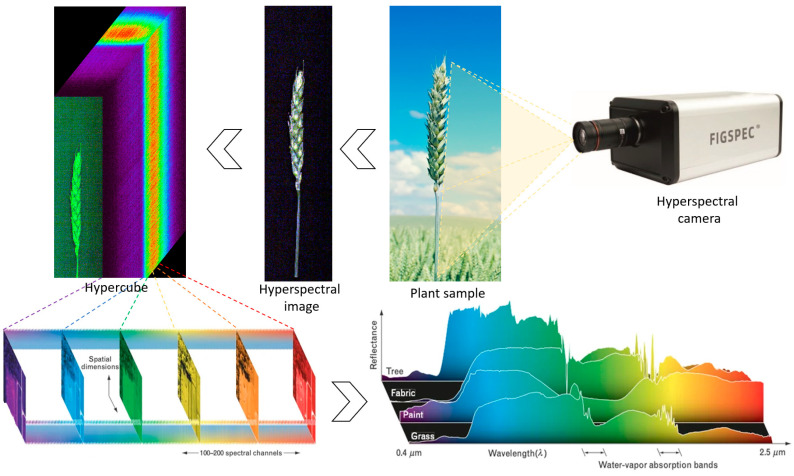
Scheme of hyperspectral data cube acquisition during hyperspectral imaging of plant samples [73].

Hyperspectral image processing and spectral extraction were performed using Breeze (licensed version 2024.2.0) and ENVI (version 5.2) with IDL scripting capabilities. Data were stored as hypercubes combining spatial and spectral information (Figure 1). Preprocessing included radiometric calibration, spatial masking, ROI extraction, smoothing, SNV normalisation, and mean-centring to enhance spectral feature discrimination.

A detailed methodology of the hyperspectral imaging procedure is provided in Appendix A.

#### 2.3.3. Interactive Spectral Analysis Using PCA and Pixel Explore

Pixel Explore was used for PCA-based spectral analysis. Selected pixels were added to the training dataset with labels. PCA scatter plots (t[1] and t[2]) helped identify main spectral differences and subtle morphological features. Max Variance Images facilitated localisation of informative regions.

#### 2.3.4. Machine Learning Algorithms for Disease Identification and Differentiation

The system was trained to detect, differentiate, and identify pathogen types by extracting relevant spectral features—such as reflectance values at specific wavelengths, vegetation indices, and statistical descriptors of spectral signatures—and combining them to create feature vectors, which were then fed into machine learning classification algorithms for disease classification:

- PCA—dimensionality reduction;
- PLS-DA—prediction of categorical variables;
- Maximum Entropy (SDCA)—logistic regression for multiclass problems;
- Neural Network (AP)—captures linear patterns;
- SVM—separates classes in high-dimensional space;
- SIMCA—PCA-based class models;
- Random Forest—ensemble of decision trees, robust to noise, evaluates feature importance [74,75,76,77,78].

Models were trained on labeled ROIs and evaluated with five-fold cross-validation using accuracy, recall, and F1-score metrics. Manual segmentation and the “Grid and Insets” function were used to mark infected regions. A total of 2184 infected regions were analysed. The software operated within a spectral range of 391.5–1006.8 nm, covering the visible and near-infrared regions [36].

**Figure 2:**
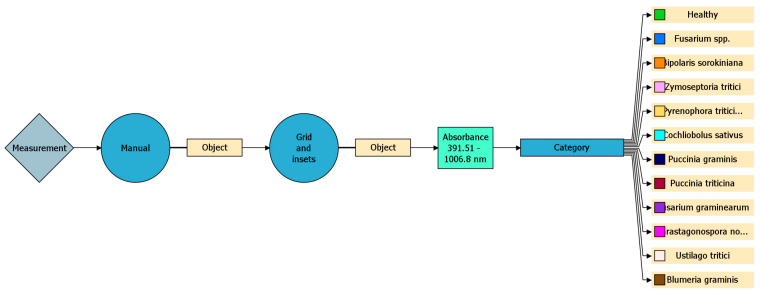
Visualisation of the decision tree with key nodes and classification criteria.

**Figure 3:**
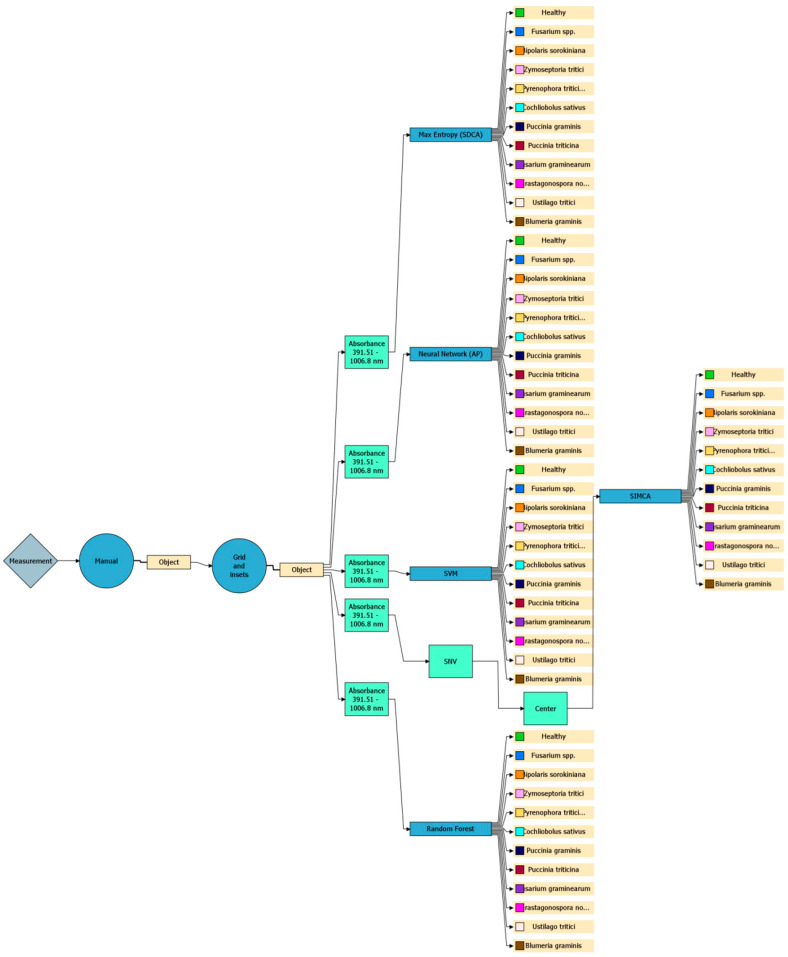
Analytical decision tree illustrating machine learning algorithms, absorption wavelength ranges, and processing parameters for disease classification.

Figure 2 and Figure 3 illustrate the workflow: decision trees with key nodes, classification criteria, wavelength ranges, and algorithmic steps.

### 2.4. Statistical Data Processing

Statistical analysis, including analysis of variance (ANOVA), was performed to validate the obtained results. These methods allow us to evaluate the reliability and accuracy of the classification outcomes. ANOVA and descriptive statistics were applied to process the spectral data.

The calculated parameters—including minimum and maximum reflectance, mean reflectance, standard deviation, and coefficient of variation—enable a quantitative assessment of the spectral characteristics of the studied objects and facilitate the identification of differences among them [79,80,81,82].

The performance of machine learning models (Precision, Recall, F1-score) for disease identification and classification was evaluated to determine the most accurate and reliable algorithm for predicting plant diseases effectively.

These metrics form the foundation for further development of classification models and can be used to enhance the accuracy and efficiency of disease identification and monitoring in spring wheat crops, ultimately improving agricultural crop protection systems.

## 3. Results

Figure 4, Figure 5, Figure 6, Figure 7, Figure 8, Figure 9, Figure 10 and Figure 11 present hyperspectral images with corresponding spectral signatures and PCA variance scatter plots for all analysed samples.

### 3.1. Spectral Characteristics

#### 3.1.1. Healthy Wheat

**Figure 4:**
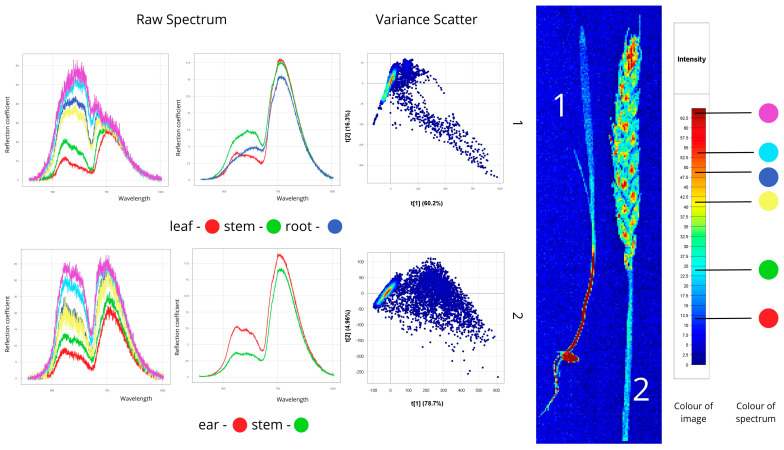
Spectral signatures of healthy plant samples revealed through hyperspectral image analysis (Raw Spectrum: *x*-axis—wavelength (nm), *y*-axis—reflectance coefficient (%); Variance Scatter: *x*-axis—principal component 1, *y*-axis—principal component 2).

Figure 4 presents hyperspectral images of healthy spring wheat, showing a young shoot (leaf, stem, root) and a wheat ear with stem.

Spectral analysis of the first sample (young shoot) showed distinct reflectance patterns among organs. The stem showed the highest reflectance (50–60%), attributable to its cellular structure and lower pigment content. The root exhibited moderate reflectance (20–40%) resulting from its morphology and lack of chlorophyll, while the leaf exhibited the lowest values (10–25%) because of strong light absorption by photosynthetic pigments. Characteristic spectral features included peaks between 500–780 nm and a pronounced “red edge” at 680–750 nm, indicating active photosynthesis.

For the second sample, the stem showed reflectance of 10–25%, while the ear reached 30–35%. The ear’s higher reflectance correlates with its biochemical composition and lower chlorophyll concentration in maturing tissues. The spectra exhibited characteristic photosynthetic features: a chlorophyll absorption dip at 640–670 nm (“chlorophyll minimum”) and a sharp reflectance rise at 680–750 nm (“red edge”). The ear demonstrated higher overall reflectance (18) than the stem (17), consistent with optical differences between reproductive and vegetative organs during heading.

Variance Scatter plot revealed compact spectral clustering with low dispersion, confirming physiological uniformity and absence of latent stress in healthy plants.

#### 3.1.2. Root Rot

**Figure 5:**
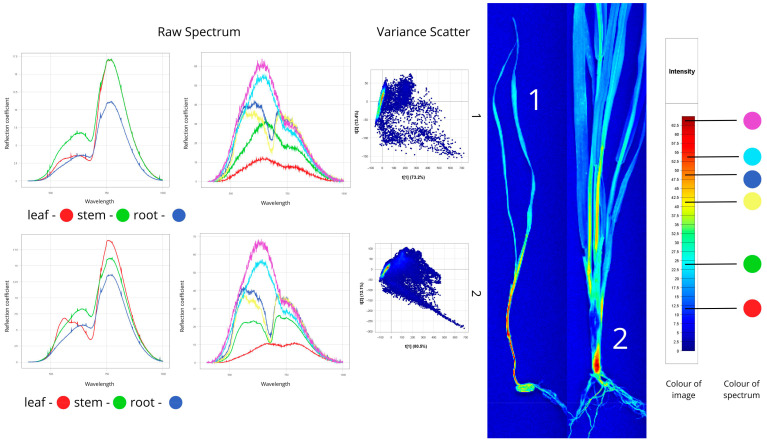
Spectral signatures of plants affected by root rot, based on hyperspectral imaging: 1-*Fusarium* spp., 2-*Bipolaris sorokiniana* (Raw Spectrum: *x*-axis—wavelength (nm), *y*-axis—reflectance coefficient (%); Variance Scatter: *x*-axis—principal component 1, *y*-axis—principal component 2).

Figure 5 shows hyperspectral images of plants affected by root rot: the first sample has fusarium root rot (caused by *Fusarium* fungi, which degrade the root’s vascular system), and the second shows helminthosporium root rot (caused by *Bipolaris* fungi, mainly attacking stem bases and root collars). Healthy leaf and stem regions in both samples exhibited high reflectance, especially near the roots (40–60% in the first sample, 40–65% in the second), reflecting intact cellular structures and normal tissue hydration. Leaves show reflectance values of 20–35%, indicating active photosynthesis and intact pigments, while roots showed low reflectance (~10%), indicating severe tissue damage and altered light reflection. Spectral peaks fall between 550–780 nm. A characteristic plateau appears in the “red edge” region (680–750 nm), suggesting reduced photosynthetic activity in diseased plants.

The root spectra of both samples have significantly lower reflectance (11–13%) compared to other parts, due to necrosis, tissue density changes, and altered moisture content. Peaks lie mostly between 550–750 nm. In the blue-green region of the spectrum (450–550 nm), an abnormal increase in reflectance is observed—a typical indicator of plant stress.

In the Variance Scatter plot, the first sample’s data points cluster compactly in the upper left sector, indicating moderate variation typical of Fusarium root rot with relatively uniform root damage. The second sample’s scatter is more elongated with greater spread, reflecting the heterogeneous nature of helminthosporium infection, where localised necrotic lesions vary in severity. This difference in scatter plot shapes enables differentiation of root rot types based on spectral feature distribution.

#### 3.1.3. Leaf Spot

**Figure 6:**
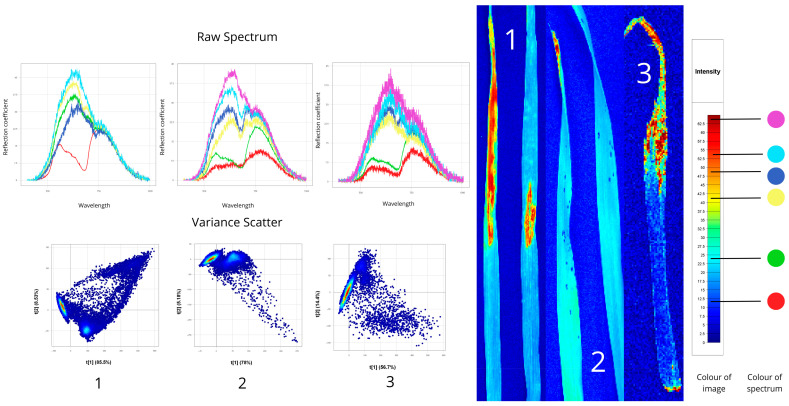
Spectral features of plant samples affected by leaf spot diseases, revealed through hyperspectral imaging: 1-*Pyrenophora tritici-repentis*, 2-*Cochliobolus sativus*, 3-*Zymoseptoria tritici* (Raw Spectrum: *x*-axis—wavelength (nm), *y*-axis—reflectance coefficient (%); Variance Scatter: *x*-axis—principal component 1, *y*-axis—principal component 2).

Figure 6 depicts leaf spot diseases: the first sample is infected with *P. tritici-repentis* (causing chlorotic and necrotic spots), the second with *C. sativus* (affecting all above-ground parts), and the third with *Z. tritici* (showing brown necrotic spots with pycnidia).

Tan spot infection caused elevated reflectance (37.5–47%) compared to healthy tissue (22.5–33%), with particularly high values in the 500–650 nm range indicating chlorophyll degradation and chlorosis. Some affected areas showed additional peaks near 750 nm, suggesting tissue lightening. In contrast, septoria tritici blotch exhibited substantially lower reflectance (10%) due to necrosis and dark pigment accumulation, while spot blotch showed wide reflectance variability (22.5–40%) reflecting heterogeneous lesion development from early chlorosis to complete necrosis. All three diseases shared common spectral characteristics, including peaks at 550–780 nm and 15–25% NIR reflectance reduction (750–900 nm), indicating mesophyll structural damage. Tan spot was further distinguished by a red-shifted peak at 680–720 nm, characteristic of photosynthetic disruption.

PCA revealed disease-specific clustering patterns. Tan spot showed distinct L-shaped distribution with high t[1] variance (86.8%), clearly separating healthy (high values) and infected (low values) tissues. Spot blotch exhibited the greatest spectral spread with strong t[1]–t[2] correlation (78% and 5.18%), reflecting concurrent disease stages from chlorosis to necrosis. Septoria tritici blotch formed triangular clusters with lower t[1] contribution (56.7%) and higher t[2] influence (14.4%), indicating complex lesion heterogeneity.

Spectral signatures provided additional differentiation: tan spot’s smooth red-region rise, spot blotch’s deep chlorophyll absorption dip (670–680 nm), and septoria’s dual peaks at 550 nm and 780 nm. These distinct patterns enable reliable early-stage disease identification using hyperspectral methods.

#### 3.1.4. Brown Rust

**Figure 7:**
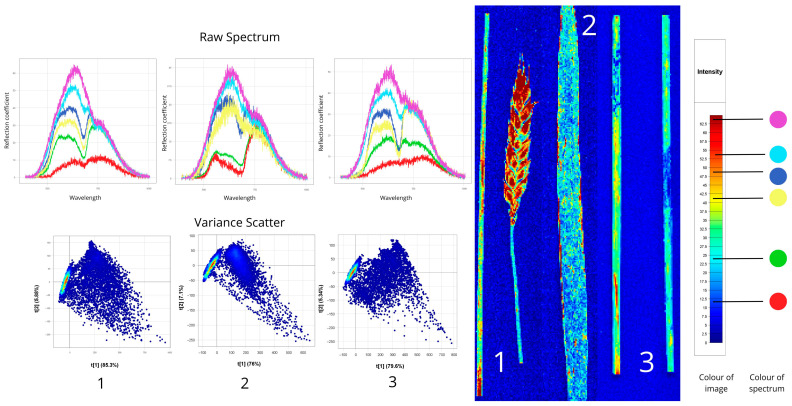
Spectral characteristics of plant samples affected by brown rust, identified through hyperspectral image analysis: 1, 3-*Puccinia graminis*, 2-*Puccinia triticina* (Raw Spectrum: *x*-axis—wavelength (nm), *y*-axis—reflectance coefficient (%); Variance Scatter: *x*-axis—principal component 1, *y*-axis—principal component 2).

Three rust types were analysed: stem rust (*P. graminis*) on ear and stem; leaf rust (*P. triticina*); and stem rust with elongated pustules. Severely infected ear tissues showed low reflectance (~10) due to dense sporulation and necrosis, while moderately infected areas reached 23–30. Healthy ear tissue showed 50–60% reflectance, representing a 5–6-fold reduction compared to diseased tissue. Spectral peaks occurred at 550–750 nm (Figure 7).

Leaf rust exhibited reflectance variation (7.5–20) reflecting scattered lesions and mosaic infection patterns. Moderately infected zones showed 22.5–45 reflectance. A characteristic reflectance dip at 600–680 nm indicated urediniospore absorption, with primary peaks at 550–750 nm. Stem rust displayed higher reflectance (30–50) due to orange-brown urediniospores. Advanced stages showed values near 10, indicating transition to dark teliospores. Infected areas exhibited spectral shifts toward NIR (550–780 nm), suggesting structural tissue changes. Ear rust showed lower reflectance than stem rust due to deeper infection in compact spikelets. Leaf rust exhibited greatest spectral dispersion in PCA, reflecting heterogeneous pustule distribution versus more focused stem infections.

#### 3.1.5. Fusarium Head Blight of Wheat

**Figure 8:**
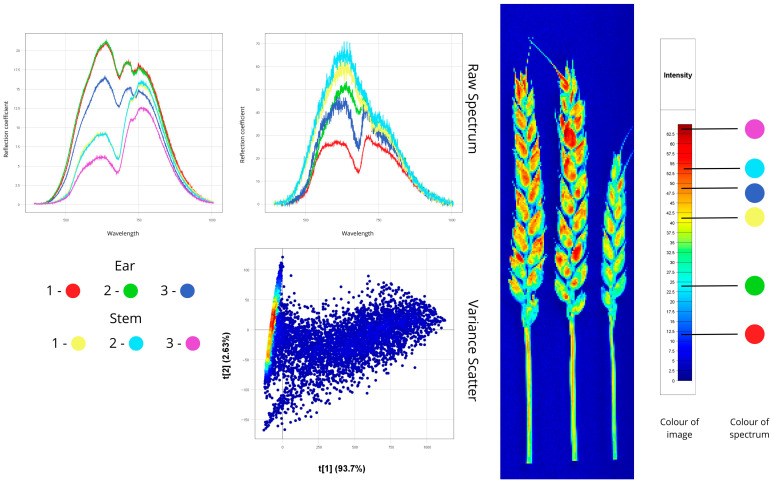
Spectral characteristics of plant samples infected with fusarium head blight (*Fusarium graminearum*), identified through hyperspectral image analysis (Raw Spectrum: *x*-axis—wavelength (nm), *y*-axis—reflectance coefficient (%); Variance Scatter: *x*-axis—principal component 1, *y*-axis—principal component 2).

Figure 8 displays wheat ears infected with *F. graminearum* during flowering and grain filling stages. The fungus produced light-coloured mycelium and spores with high reflectance (60–70%) in the 500–600 nm range, while surrounding chlorotic zones showed 40–50% reflectance. This contrasted with healthy tissues (~30% reflectance), demonstrating that fungal colonisation and chlorophyll degradation increase visible-light reflectance.

Spectral peaks for infected parts range from 550 to 750 nm, with a pronounced peak in the orange-red range (600–650 nm), typical for bleached tissues. Dried and healthy tissues have peaks from 600 to 750 nm. This shift of spectral peaks toward shorter wavelengths in diseased tissues is a diagnostic marker for fusarium head blight, helping distinguish it from other ear diseases.

Infected ears exhibited higher reflectance than stems, a characteristic feature of Fusarium infection resulting from replacement of dark pigments by light fungal structures. Reflectance intensity directly correlated with disease severity, showing a gradient from least to heavily infected samples. Spectral peaks occurred at 600–780 nm, while NIR reflectance (750–900 nm) decreased by 15–20% versus healthy tissue, indicating cellular structural damage.

The Variance Scatter plot shows a compact point distribution with no sharp changes. The homogeneity of spectral data suggests relatively uniform Fusarium development within each ear, without sharp local variations. Importantly, no significant outliers are present, confirming the synchronous development of Fusarium across the ear and resulting in a homogeneous spectral signature.

#### 3.1.6. Septoria Glume Blotch of Wheat

**Figure 9:**
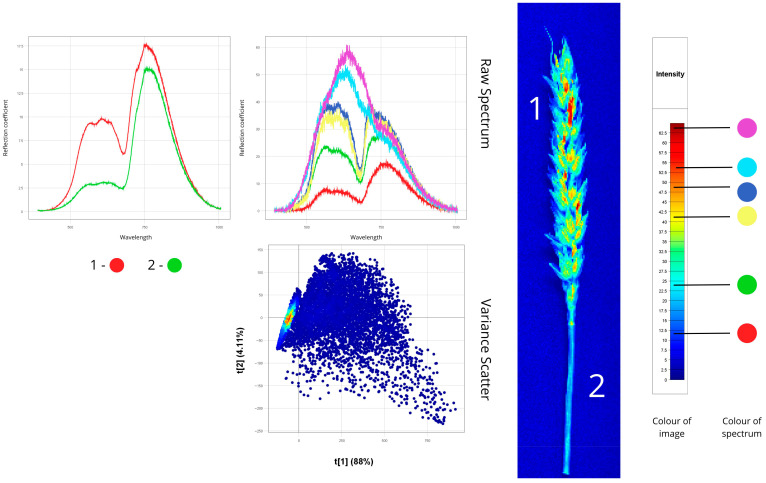
Spectral characteristics of plant samples affected by septoria glume blotch (*Parastagonospora nodorum*), identified through hyperspectral image analysis (Raw Spectrum: *x*-axis—wavelength (nm), *y*-axis—reflectance coefficient (%); Variance Scatter: *x*-axis—principal component 1, *y*-axis—principal component 2).

Septoria glume blotch (*P. nodorum*) showed very low reflectance (10–15%) due to dark necrotic spots and pycnidia. Adjacent chlorotic zones exhibited high reflectance (50–60%) from chlorophyll and carotenoid degradation, while healthy tissues reflected 25–35%. This created clear spectral contrasts between disease stages within individual wheat ears (Figure 9).

Characteristic spectral features included a pronounced dip in the 680–710 nm range for infected areas, reflecting complete destruction of the photosynthetic apparatus. Chlorotic zones show unusually high reflectance in the green spectral region (550–570 nm).

Overall, ears showed higher reflectance (17.5%) compared to stems (15%). This paradoxical increase is explained by the dominant contribution of extensive chlorotic zones masking the presence of dark necrotic spots. The spectral wavelength range for these areas averages between 550 and 750 nm. In the near-infrared range (780–900 nm), reflectance decreases by about 20–25% compared to healthy ears, likely due to profound structural tissue changes.

The Variance Scatter plot showed reduced homogeneity versus healthy plants, with an elongated point cloud reflecting combined necrosis and chlorosis effects. Visible subclusters corresponded to different disease stages, from initial chlorosis to sporulating necrosis. The spread along t[1] (88% variance) confirmed infection degree as the key spectral differentiator (Figure 9).

#### 3.1.7. Loose Smut of the Ear

**Figure 10:**
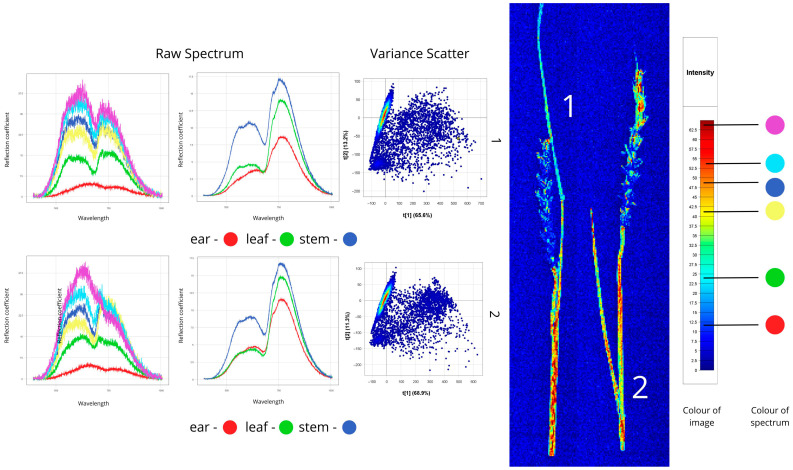
Spectral signatures of wheat samples infected by loose smut (*Ustilago tritici*), derived from hyperspectral image analysis (Raw Spectrum: *x*-axis—wavelength (nm), *y*-axis—reflectance coefficient (%); Variance Scatter: *x*-axis—principal component 1, *y*-axis—principal component 2).

Figure 10 displays two wheat ear samples: the first is fully affected by loose smut (*Ustilago tritici*), which typically infects all parts of the ear and forms a dense black spore mass; the second is only partially affected, with some healthy floral structures still intact.

Diseased areas showed extremely low reflectance (7%) due to dense dark teliospores, while adjacent bleached tissue reached 15% reflectance. Healthy parts showed 22–37.5% reflectance with spectral peaks at 550–750 nm. The characteristic “red edge” (680–750 nm) was absent in infected zones, indicating complete photosynthetic function loss.

Overall reflectance was lowest in infected ears (8–11), with higher values in leaves (13–15) and stems (17.5). Healthy tissue peaks shifted toward NIR (~780 nm), while diseased samples peaked in green region (550–570 nm).

PCA revealed strong point scattering with bimodal distribution: tight healthy cluster and dispersed disease severity cluster. This contrast provides reliable diagnostic differentiation. Dispersion along t[2] indicated simultaneous changes in multiple optical parameters, forming a distinct spectral signature for loose smut.

#### 3.1.8. Powdery Mildew

The hyperspectral image displayed a leaf infected with powdery mildew (caused by *B. graminis*). The violet and turquoise curves represent active sporulation areas, with reflectance values between 70 and 80. These abnormally high values result from the dense white fungal mycelium on the leaf surface, which effectively scatters light across the visible spectrum. Surrounding partially infected areas show reflectance near 50, representing early mycelial development with some remaining leaf structure intact. The remaining leaf areas showed 10–30% reflectance. This strong contrast—often exceeding 60 units—between healthy and diseased tissue is a diagnostic hallmark for powdery mildew.

**Figure 11:**
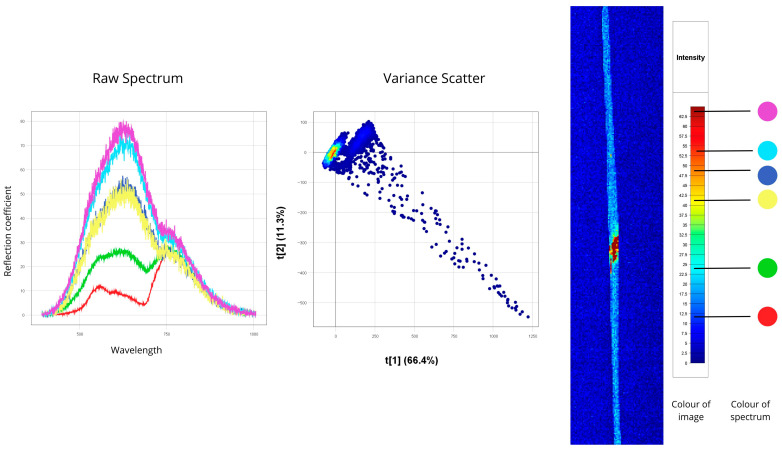
Spectral signatures of plant samples affected by powdery mildew (*Blumeria graminis*), identified through hyperspectral image analysis (Raw Spectrum: *x*-axis—wavelength (nm), *y*-axis—reflectance coefficient (%); Variance Scatter: *x*-axis—principal component 1, *y*-axis—principal component 2).

Spectral peaks occur in the 550–750 nm range. In the “red edge” region (680–720 nm), infected zones show a noticeably flattened peak, reflecting disruption of the photosynthetic machinery, while healthy tissue retains a sharp rise. The Variance Scatter displays a strongly elongated cluster, with a slope exceeding 45°, indicating simultaneous shifts across brightness, spectral shape, and peak positions. The white fungal coat alters surface reflectance and increases spectral variability, resulting in widely dispersed data points. The first principal component t[1] accounts for 66.4% of the total variance, underscoring the dominant influence of the disease on the plant’s optical response. Moreover, multiple subclusters in the scatter reflect different developmental stages of disease—from initial mycelial growth to full sporulation features (Figure 11).

Thus, powdery mildew produces a distinctive spectral signature with extremely high reflectance values (about 70–80), and the clustering pattern in the scatter plot enables highly accurate detection across disease stages.

### 3.2. Statistical Analysis

Statistical analysis revealed several key patterns in the spectral data of plant tissues affected by disease. These trends relate both to variability in spectral response and to the resilience of specific plant parts. Key spectral parameters included wavelength ranges where light is absorbed and reflected, as well as the reflectance coefficients of the observed tissues (Table 1).

**Figure 12:**
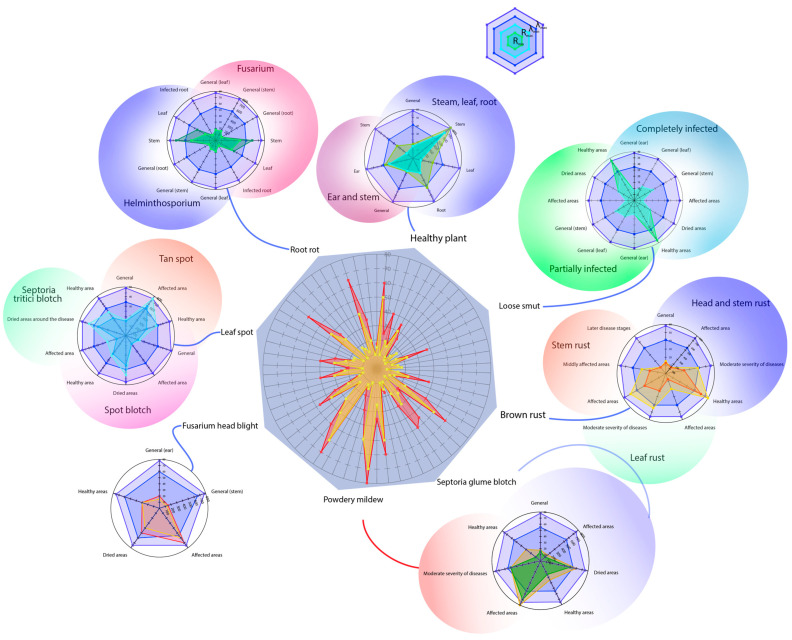
Spectral parameters characterising the reflectance and absorption properties of spring wheat samples (healthy and infected by pathogens of various aetiologies).

As shown in Figure 12, reflectance analysis revealed that the highest values were recorded in areas affected by powdery mildew (70–80%), primarily due to the presence of light-coloured fungal mycelium that strongly scatters light. In contrast, the lowest reflectance values were observed in tissues affected by loose smut (7%) and spot blotch (10%). This resulted from high light absorption by dark pigments and dense spore masses within the infected zones. Reflectance in healthy tissues varies depending on the plant organ: the stem showed the highest values (50–60%), while the leaf had the lowest (10–25%). For roots, reflectance ranged from 20 to 40%, with these differences attributed largely to the unique biochemical composition and structure of each plant organ.

The main reflectance peak across all samples fell within the 550–780 nm range. However, in diseased tissues, the position of this peak often shifts. For example, in fusarium-infected wheat ears, the peak moves toward the 600–780 nm range, while healthy tissues retain a strong near-infrared peak between 720 and 780 nm. Moreover, the typical “red edge” (680–750 nm)—a hallmark of photosynthetically active vegetation—is well defined in healthy plants but flattened or missing in infected samples, indicating impaired photosynthetic function (Figure 12).

**Figure 13:**
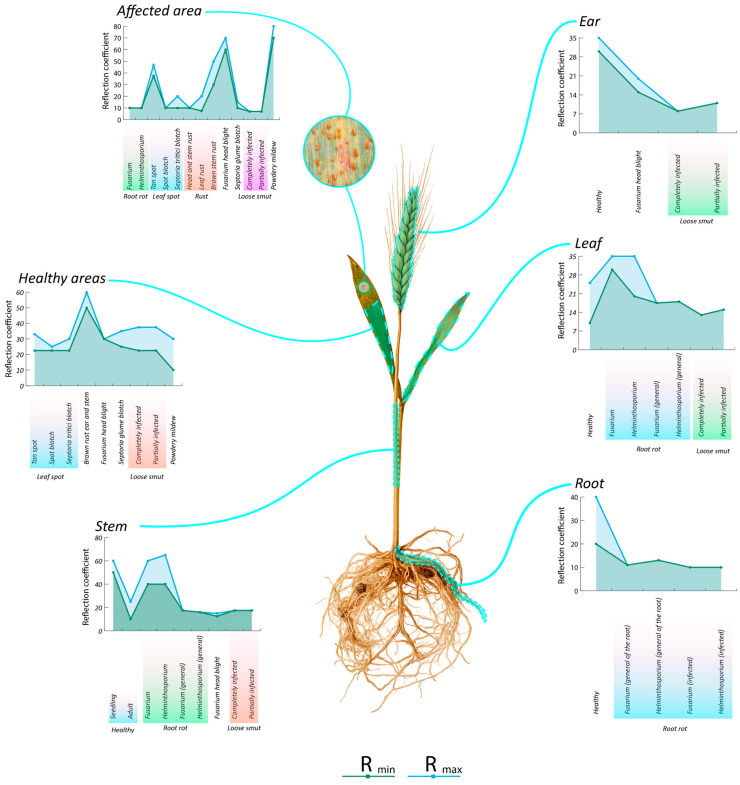
Reflectance values of various spring wheat organs under different disease conditions (*x*-axis—type of infection/disease/degree of damage; *y*-axis—reflectance coefficient, nm).

A comprehensive spectral analysis (Figure 13) demonstrates consistent patterns in reflectance values across different diseases, each driven by distinct physiological and structural alterations in the plant.

The highest polarisation in reflectance was found in wheat ears, especially under fusarium head blight and powdery mildew infection (60–80%), due to the formation of light-coloured mycelial structures, chlorophyll degradation, and changes in surface microstructure that enhance light scattering. On the other hand, loose smut and septoria glume blotch exhibited extremely low reflectance values (7–15%), primarily due to melanin-rich spores, necrosis, and dense spore layers that absorb light.

Leaves exhibited the widest reflectance range (7.5–80%), reflecting the diversity of pathological processes: chlorosis and pigment degradation increase reflectance, whereas necrosis and tissue darkening decrease it. Additionally, the biological characteristics of specific pathogens play a major role—for instance, epiphytic fungi (e.g., powdery mildew) tend to cause high reflectance, while endophytic or necrotrophic fungi typically result in lower values.

Compared to foliar tissues, the root system exhibited a narrower reflectance range (10–40%), with a marked decrease under infection (~10%). This likely results from tissue maceration, degradation, accumulation of metabolic byproducts, and changes in moisture and density. In contrast, the stem demonstrated relative spectral stability, maintaining reflectance values of 40–65% even under disease pressure. This change can be attributed to its anatomical features, such as a waxy cuticle, dense cellular structure, and relatively low chlorophyll content (Figure 13).

These spectral differences are underpinned by three primary biophysical mechanisms: light scattering by pale fungal structures (e.g., mycelium); absorption by dark pigments, particularly during chlorophyll breakdown and melanin accumulation; microstructural alterations of the plant surface affecting reflectance behaviour.

The established relationships between disease type and spectral response form the basis for the development of multiparametric diagnostic models. These models can leverage not just absolute reflectance values but a broader spectrum of characteristics—including variability across spectral bands and distribution patterns—to enable more accurate detection and classification of plant diseases.

### 3.3. Classification Model: Machine Learning Algorithms

While previous studies explored hyperspectral imaging for detecting root rot [83], leaf spot diseases [84], rust [85], fusarium head blight [86], septoria glume blotch [53], and powdery mildew [87], our work takes a comprehensive approach by considering multiple specific wheat diseases together, with the ability to differentiate between various pathogen types and infection stages.

In this study, we developed a classification model based on spectral profiles for the diseases examined. A total of 2184 hyperspectral wheat samples were analysed, divided into 11 categories—including healthy plants and infections caused by different pathogens. The largest proportion of samples were infected with *F. graminearum* (37.7%), *P. graminis* (13.8%), and *P. nodorum* (13.9%). The dataset also included healthy samples (57 samples, 2.61%), *Fusarium* spp. (32, 1.47%), *B. sorokiniana* (56, 2.56%), *Z. tritici* (267, 12.2%), *P. tritici-repentis* (74, 3.39%), *C. sativus* (87, 3.98%), *P. triticina* (134, 6.14%), *U. tritici* (33, 1.51%), and *B. graminis* (15, 0.687%).

Given the imbalanced distribution of samples across classes, certain observations merit further discussion. Notably, *B. graminis* samples represented only a small fraction of the dataset (0.687%, *n* = 15). The high F1 score observed for Random Forest in this class may be influenced by the limited sample size. Specifically, Random Forest can sometimes achieve seemingly high performance for small classes if the few available samples are easily separable or are overrepresented in certain trees, which can lead to an optimistic estimate of its effectiveness. In contrast, algorithms such as Maximum Entropy and SIMCA, which rely on global data distributions and probabilistic patterns, are less prone to such inflation. Therefore, while the metrics indicate good classification performance, caution should be exercised when interpreting results for classes with very limited samples. This highlights the need for further validation with larger datasets to ensure the robustness of the model across all disease classes.

For such imbalanced datasets, it’s crucial that the model can reliably distinguish even the less represented classes. We trained models using five different algorithms: Maximum Entropy (SDCA), Neural Network (AP), Support Vector Machine (SVM), Random Forest, and SIMCA. Table 2 summarises their performance based on the R^2^Y and Q^2^Y metrics.

According to the table, SIMCA demonstrates higher R^2^Y values (indicating explained variance of the dependent variable) and Q^2^Y values (indicating predictive performance) compared to Random Forest. This is because SIMCA is more robust to class imbalance by building separate PCA-based models for each class. In contrast, Random Forest combines multiple decision trees to classify all classes simultaneously, focusing primarily on overall classification accuracy. The Maximum Entropy (SDCA) model demonstrated limited accuracy due to its linear nature, which struggles to capture the nonlinear spectral differences. The Neural Network (AP) requires large and balanced datasets for effective training, whereas SVM has difficulty distinguishing classes with substantial spectral overlap and is sensitive to kernel parameter selection.

In addition, the performance of each algorithm (except SIMCA) was evaluated using four additional classification metrics commonly used in machine learning, as shown in Table 3. These metrics include Macro Accuracy (average accuracy across classes), Micro Accuracy (accuracy across all samples regardless of class), Macro Accuracy Test (generalisation ability on new data), and Log Loss with Log Loss Reduction (measuring the quality of probabilistic predictions and improvement over a baseline model).

This analysis revealed significant differences in the effectiveness of the four machine learning algorithms. Random Forest achieved the best performance, with the lowest Log Loss, highest Log Loss Reduction, and highest Micro Accuracy, indicating strong classification accuracy and learning capability. However, its decreased Macro Accuracy Test suggests some degree of overfitting on newly labelled data. Maximum Entropy (SDCA) offers a balanced solution with consistently high scores across metrics and good generalisation ability, making it a reliable choice for practical applications. SVM performed moderately, showing acceptable but not optimal generalisation on test data. The Neural Network (AP) model requires larger datasets or more refined architectures for improved learning effectiveness.

Despite the statistical data discussed above, the most informative evaluation comes from the classification accuracy and the results of the confusion matrix.

**Figure 14:**
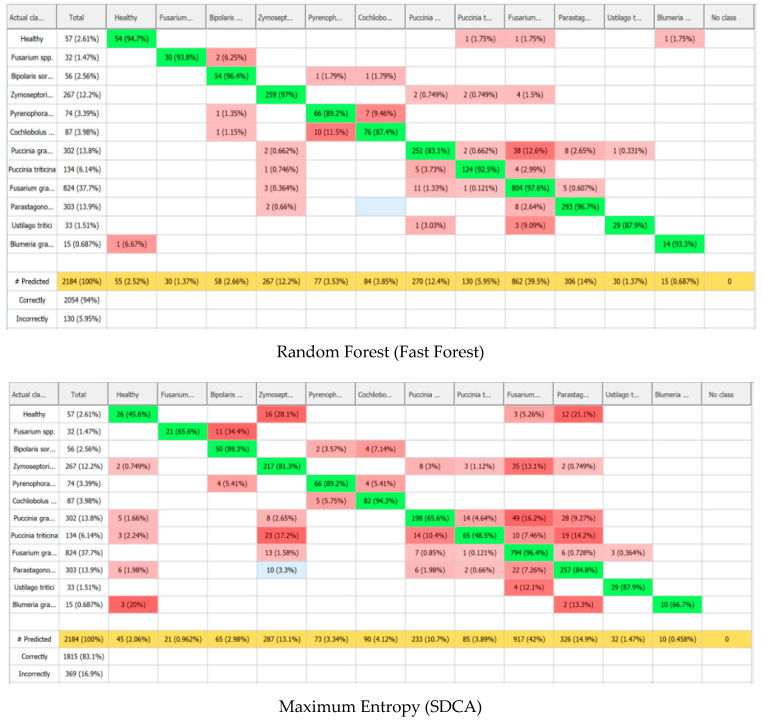

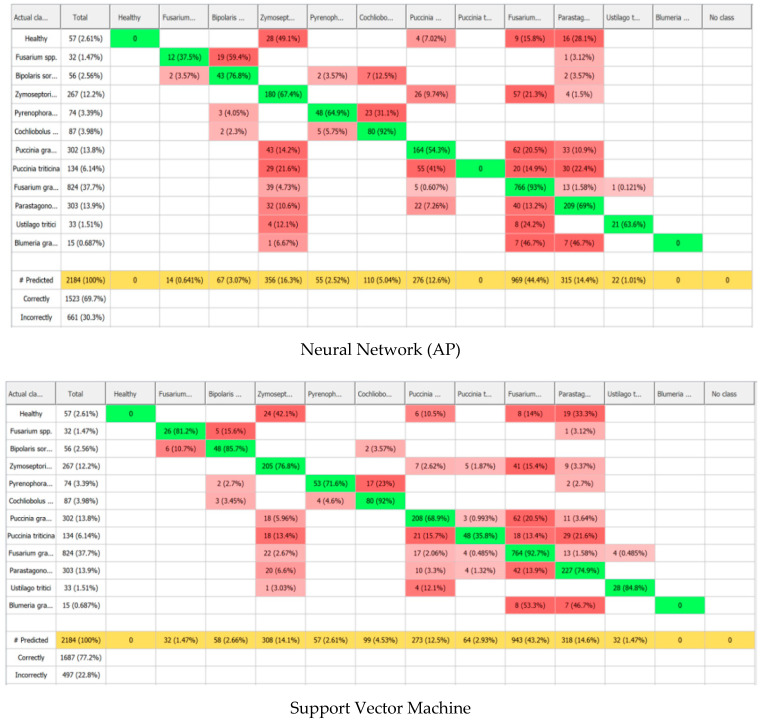
Confusion matrix for disease classification using Random Forest, Maximum Entropy, Neural Network, and SVM methods (green—correctly classified samples; bright red—largest proportion of incorrectly classified samples; pale red—smallest proportion of incorrectly classified samples).

As shown in Figure 14 and Table 4, the Random Forest algorithm achieved the highest sample identification performance. Out of 2184 samples, 2054 were correctly classified, corresponding to an overall accuracy of 94%. Only 130 samples (5.95%) were misclassified—an impressively low error rate, especially considering the high biological variability of the dataset. The largest class, *F. graminearum*, was identified with an accuracy of 97.6%, confirming the model’s robustness for large sample groups. The model also performed well in distinguishing between diseases with similar spectral profiles. For instance, *Z. tritici* and *P. nodorum* were identified with 97% and 96.7% accuracy, respectively. *P. triticina* (92.5%) and *P. graminis* (83.1%) were also reliably classified, despite some minor misclassifications likely caused by overlapping spectral features. Moderate classification errors were observed between *Cochliobolus* spp. (87.4%) and *Pyrenophora* spp. (89.2%), possibly due to shared visual symptoms such as similar types of leaf spotting. Even with smaller class sizes, the model maintained high accuracy for under-represented classes like *B. graminis* and *U. tritici* (87–93%), highlighting Random Forest’s ability to handle imbalanced datasets effectively.

Occasional misclassifications—for example, samples with fusarium head blight or powdery mildew being labelled as healthy—can likely be attributed to similar spectral curves and reflectance levels. A higher error rate was also noted between disease types with similar presentations. For instance, *P. tritici-repentis* was sometimes misclassified as *B. sorokiniana*, and vice versa. This may stem from overlapping symptoms, such as chlorotic halos around necrotic lesions, leading to closely resembling spectral profiles.

A similar issue was seen with *P. graminis*, which was occasionally misidentified as *F. graminearum*. These cases are likely due to shared spectral signatures, especially in early infection stages where chlorophyll content and tissue structure are similarly altered.

**Figure 15:**
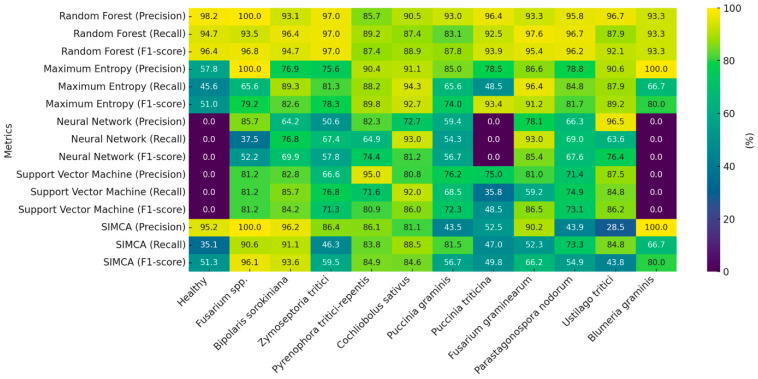
Summary map of evaluation metrics for machine learning algorithms.

According to the confusion matrix, Precision (the proportion of correct positive predictions) ranged from 85.7% to 100%, Recall (the ability to correctly identify all true positives in a class) ranged from 83.1% to 97.6%, and the F-score (the harmonic mean of precision and recall) ranged from 87.4% to 97% (Table 4 and Figure 15). The Maximum Entropy (SDCA) method showed moderate capability in differentiating between classes. It achieved the highest accuracy when classifying *C. sativus* and *F. graminearum* (94.3–96.4%), likely due to the distinct spectral features associated with these diseases—such as chlorophyll degradation and necrosis in the former and high reflectance values in the latter. Conversely, the model struggled to accurately classify healthy plants and samples infected with *P. triticina* (45.6–48.5%). Notably, this lower accuracy wasn’t directly tied to sample size, indicating the method’s sensitivity to spectral similarity between classes rather than class imbalance. In this case, healthy tissue and leaf rust may share similar spectral characteristics due to the high reflectivity of healthy leaves and the presence of chlorosis around infection sites (Table 4, Figure 14 and Figure 15).

The Neural Network (AP) and SVM models delivered the weakest performance overall. Both failed entirely to correctly classify healthy plants and *B. graminis* samples, suggesting overfitting and a limited ability to detect subtle spectral differences. In addition, the Neural Network often misclassified *P. graminis* samples, possibly due to high spectral variability caused by heterogeneous lesion development. Although both models achieved relatively high accuracy for *F. graminearum* (92.7–93%)—the largest class—their overall effectiveness remained low. This highlights that a large dataset alone is not enough if the algorithm cannot accurately capture the complex spectral relationships in the data (Table 4, Figure 14 and Figure 15).

**Figure 16:**
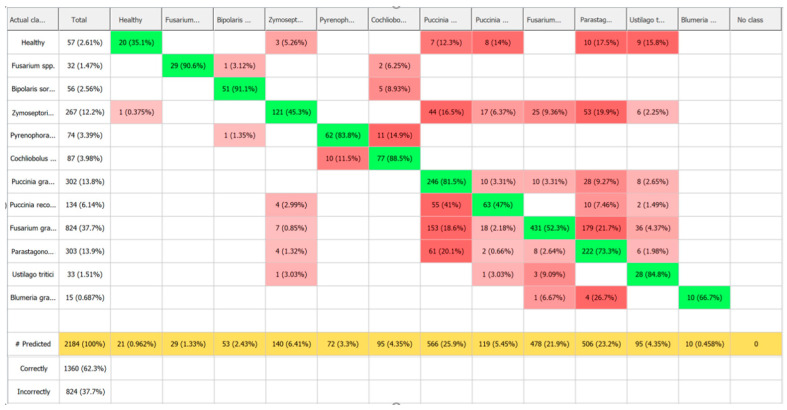
Confusion matrix for disease classification using SIMCA method (green—correctly classified samples; bright red—largest proportion of incorrectly classified samples; pale red—smallest proportion of incorrectly classified samples).

The SIMCA model, based on multivariate statistics rather than machine learning, performed best in identifying *Fusarium* spp. (90.6%) and worst when classifying healthy samples (35.1%), which lack distinct spectral features. Unlike machine learning methods, SIMCA builds individual models for each class, making it more sensitive to within-class variability but less resilient when spectral features overlap between different pathogens (Table 4, Figure 15 and Figure 16).

Some results are reported as 0 because certain machine learning algorithms were unable to classify or detect specific plant disease lesions. This occurred due to insufficient distinguishing features in the input data, specifically for a certain ML algorithm, overlapping spectral characteristics of healthy and diseased tissue, or limitations in the training dataset for these particular disease types (this may be mainly due to individual features of machine learning algorithms).

**Figure 17:**
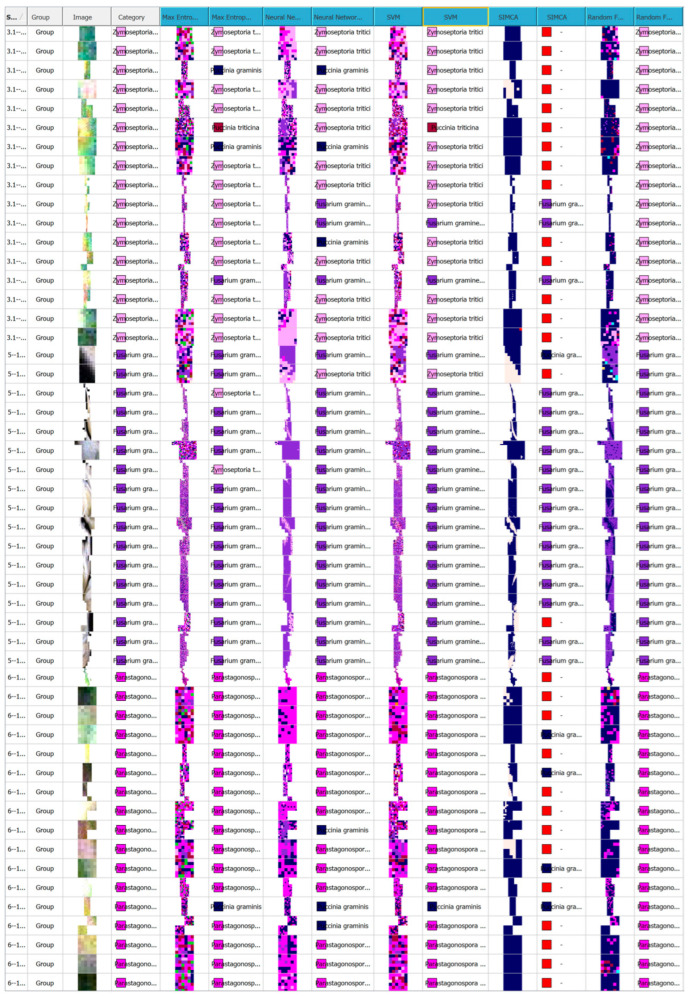
Comparative results of classification models for detecting wheat diseases (the blue line at the top reflects the classification results obtained using machine-learning methods: visual representation of the area and identification of the disease pathogen).

More detailed classification results for each algorithm are presented in Figure 17.

In conclusion, Random Forest operates as an ensemble of decision trees, each trained on a random subset of features. This enables the model to effectively capture spectral patterns—such as combined reflectance in the near- and mid-infrared ranges—that linear models fail to represent. It is also highly robust to noise in hyperspectral images caused by lighting conditions or other external factors, as predictions are averaged across hundreds of trees, minimising the influence of outliers and random errors. In contrast, models like Neural Networks and SVM are more prone to overfitting when trained on small or imbalanced datasets and often struggle to generalise without extensive parameter tuning. Random Forest also provides insight into feature importance, allowing researchers to evaluate which wavelengths contribute most to the classification process—an essential capability in hyperspectral disease diagnostics.

## 4. Discussion

### 4.1. Interpretation of Statistical Indicators

**Figure A1:**
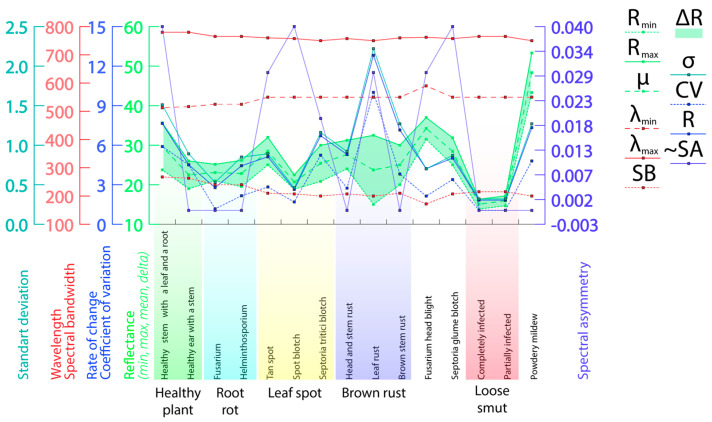
Average spectral indicators for different parts of spring wheat samples (healthy and diseased).

The results of key statistical metrics, presented in Table A1 (Figure A1), reveal consistent patterns and correlations between various physiological and spectral characteristics of wheat plants. A clear relationship was observed between the mean reflectance coefficient (*μ*) and the coefficient of variation (CV). For instance, powdery mildew—associated with the highest reflectance values (48.33%)—also showed one of the highest CVs (4.8%), suggesting a direct link between spectral response heterogeneity and the severity of infection. A similar trend was evident in the case of brown rust.

An inverse relationship was identified between the reflectance coefficient and the spectral bandwidth (SB). Healthy samples exhibited moderate reflectance values (22.50–29.69%) with the broadest spectral ranges (263–268 nm). In contrast, fusarium head blight showed elevated reflectance (34.25%) with significantly narrower SB (172 nm)—indicating a disruption of the plant’s normal spectral profile due to disease processes. Interestingly, diseases characterised by low reflectance values tend to show relatively stable spectral patterns. For example, loose smut (*μ* = 15.08–15.92%) is marked by minimal variation (CV = 1.06%) and a low standard deviation (*σ* = 0.32), suggesting consistent and uniform pathological changes associated with this disease.

A particularly distinctive diagnostic marker is the combination of a shift in the minimum wavelength (to 590 nm) and a narrowing of the spectral bandwidth (SB = 172 nm), as seen in fusarium head blight. This specific spectral signature can serve as a reliable differentiator from other types of wheat diseases.

Brown leaf rust exhibited the widest spread in reflectance values (*R* = 12.83), indicating a highly heterogeneous pattern of disease progression, ranging from early-stage symptoms to complete tissue necrosis. A similar mosaic progression pattern occurred in powdery mildew and septoria, reflected in their high reflectance variability (*R* = 7.33 and 6.72, respectively). Conversely, minimal reflectance variability is seen in loose smut (*R* = 1.83) and Fusarium root rot (*R* = 2.78), likely due to the synchronised development and uniformity of disease symptoms. A narrowing of the spectral bandwidth in comparison to healthy plants indicates disrupted metabolic processes, a feature commonly associated with septoria tritici blotch, spot blotch (SB = 200 nm and 208 nm, respectively), and brown rust (SB = 200–210 nm). Heterogeneous or uneven disease manifestation may result in spectral asymmetry- a parameter that offers further insight into the nature of infection. Approximate asymmetry calculations help differentiate between disease types. For example, spot blotch (~SA = 0.04) shows pronounced asymmetry due to the alternating presence of necrotic and chlorotic lesions, creating a visibly patchy pattern. septoria glume blotch also exhibits similar asymmetry levels, pointing to the uneven distribution of necrotic areas and pycnidia. Brown rust and tan spot show slightly lower asymmetry values (~0.03), reflecting the irregular emergence of pustules and chlorotic spots. A slightly lower but still detectable asymmetry (~0.02) characterises septoria tritici blotch. In contrast, no significant asymmetry is observed in diseases like powdery mildew, fusarium infections, loose smut, or stem rust. This indicates a uniform distribution of fungal mycelium, systemic infection patterns, and synchronised pustule formation across affected areas. Interestingly, a healthy young plant sample also showed asymmetry (~0.04), likely due to natural variations in chlorophyll distribution or anatomical differences between plant organs. As such, spectral asymmetry appears to be a highly informative parameter for distinguishing between diseases with mosaic symptom patterns (like septoria tritici blotch and spot blotch) and those with systemic or uniform pathology (like loose smut and fusariosis).

Overall, the spectral data collected enable clear differentiation of wheat diseases based on distinct optical signatures. For example, high reflectance values (>60%) in diseases like fusarium head blight and powdery mildew are related to the light-scattering properties of fungal mycelium and spores. In contrast, darkening of plant tissues—seen in diseases such as septoriosis, spot blotch, and loose smut—results in low reflectance values (<15%). Chlorotic symptoms and healthy tissues typically reflect light within a moderate range (20–50%). These diagnostic thresholds form a solid foundation for developing robust algorithms to enable automated disease detection—critical for precision agriculture and crop health monitoring systems.

### 4.2. Spectral Characteristics of Various Wheat Diseases

#### 4.2.1. Healthy Areas

**Figure A2:**
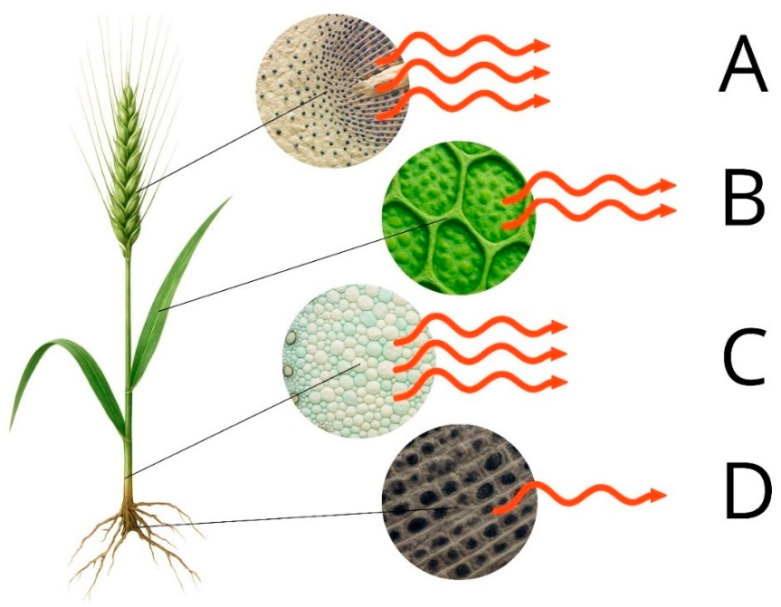
The intensity of light reflection from different parts of plants ((**A**)—spike (highest reflectance—three arrows), (**B**)—leaf (medium reflectance—two arrows), (**C**)—coleoptile (high reflectance—three arrows), (**D**)—root (low reflectance—one arrow)).

Different parts of healthy wheat plants showed only minor differences in reflectance coefficients. Healthy wheat spikes contained silky bracts (both glumes and floral husks) and numerous air spaces within the tissues, all of which contribute to multiple scatterings of light from its smooth, dry surface. As a result, the spike shows the highest reflectance intensity among all plant parts. The healthy leaf, rich in green chlorophyll, also reflects light—although slight local decreases in reflectance may occur where chlorophyll density is especially high or where certain leaf zones lost moisture after sampling. Coleoptiles of young cereals showed pale colouration due to development below the soil surface and contains little chlorophyll but a higher proportion of conducting tissue with transparent vacuoles and colourless compounds. These pale regions of the coleoptile may reflect light more than the leaf blade because their structural components absorb little in specific spectral regions and instead largely reflect light. Meanwhile, the rest of the stem shows reflectance intensity similar to that of the leaf blade, since it likewise contains chlorophyll. The root exhibits a lower reflectance coefficient compared with the leaf and stem, owing to its darker colour (due to suberin and lignin in cell walls imparting a yellow-brown hue), phenolic compounds (such as tannins) that may accumulate in roots, as well as attached soil particles and microorganisms on the root surface that further darken the colour. On the whole, however, the differences in reflectance intensity among the spike, leaf, stem and root of a healthy spring-wheat sample are modest. Figure A2 illustrates how reflectance intensity varies across different plant parts.

#### 4.2.2. Dried Areas and Initial Stages of Diseases

As diseases progressed, different stages displayed distinct colours. Early disease symptoms often include lightening and yellowing of plant tissue—such as in rust or leaf-spot diseases. Initial signs of infection may include reduced water status and cellular dehydration [88], senescence due to membrane breakdown and reduced photosynthetic activity from chlorophyll loss [89], decreased cell-wall and membrane density, cytoplasmic changes and accumulation of reactive oxygen species (ROS). For instance, chloroplast destruction due to excess urea may lead to tissue lightening [90].

Such light-coloured zones often appear around infection sites—as seen with leaf spot diseases (for example, tan spot or septoria tritici blotch) and septoria glume blotch in wheat. Lightening caused by tissue drying and chlorophyll loss alters optical properties: less absorption and greater scattering, especially across the visible spectrum, which boosts reflectance. In hyperspectral imagery, these areas appear as isolated yellow or blue patches often adjacent to disease foci. Depending on the disease and its stage, the spectral signature varies. Diseases with weakly reflecting infected areas included loose smut and root rot, septoria glume blotch and spot blotch—these appear blue on hyperspectral images.

Conversely, diseases with high reflectance include powdery mildew, leaf spot, and fusarium head blight—these are rendered as red and orange zones on hyperspectral imagery.

#### 4.2.3. Diseases with Low Reflection

Diseases characterised by low reflectance consistently displayed darkly pigmented lesions. Root rot manifested as weakened tissue colour in infected roots, due to the accumulation of dark pigments. These pigments may play a role in the pathogen’s life cycle—facilitating tissue breakdown (as in white mould [91]), contributing to fungal survival in soil (as in black root rot pathogens [92]), or exerting toxic effects. Fungal pathogens produce hydrolytic enzymes and mycotoxins that degrade host tis-sues, disrupt water and nutrient transport, and ultimately weaken the plant. They penetrate through the epidermis and root hairs and subsequently colonize the root cortex, a process that leads to a reduced reflectance coefficient in the affected root system. While direct evidence of melanin in loose smut spores is still limited, its dark appearance may suggest similar pigment production, as has been documented in *Podospora anserina* [93], *Pestalotiopsis microspora* [94] and *Ustilago maydis* [95]. Dark colouration enhanced light absorption and reduced reflectance; hence, the spectral profile of infected organs shows markedly reduced reflectance. A similar phenomenon occurs with spot blotch (caused by fungi of the genus Helminthosporium and related species), which appears as dark spots on leaves, stems and other parts of the plant.

Rust diseases, caused by *P. graminis* fungi, spread via spores containing melanin-like pigments. Urediniospores accumulate flavonoid derivatives and carotenoids (e.g., β- and γ-carotene) [96], which protect against ultraviolet radiation and oxidative stress and give the spores their characteristic reddish hue. Teliospores likely contain melanin-related compounds, a conclusion supported by the presence of genes encoding laccase-like proteins [97] and laccase enzymes [98] in rust pathogens. Visually the disease appears as brown or dark lesions, which in hyperspectral images appear as distinct blue zones. Elevated melanin production correlates with increased fungal genome abundance in wheat roots and more severe symptoms [99]. Ear blight caused by *Septoria* and *Stagonospora* likewise produces brown lesions on spike bracts, resulting in lower reflectance of the affected area.

Darker colouration in infected areas intensifies when tissues become moist and fungal growth progresses [100]. Furthermore, pathogens destroy leaf cells, leading to necrosis and darkening of plant tissue. The reduction in reflectance due to dark pigments, sporulation and tissue degradation aligns with findings from other studies [101]. Dark-pigmented fungal diseases in plants may represent adaptations to spore dispersal and survival in field conditions. As a defensive reaction, plants may enhance melanin or phenolic compound synthesis in tissues, which also triggers darkening [102]. Melanin can protect fungal spores from UV, increase resistance to desiccation and fortify the spore wall.

#### 4.2.4. Diseases with High Reflection

Diseases characterised by whitish coatings or light-coloured lesions appeared as red or orange regions in hyperspectral imagery, reflecting strongly compared to nearby healthy tissues. These areas tend to be isolated and display higher variability in reflectance. For example, powdery mildew, caused by microscopic ectoparasitic fungi of the order *Erysiphales*, produces a white, powdery mycelium on the surface of wheat leaves. The fine, transparent, pale hyphae penetrate host cells with haustoria and coat the plant in a white layer—often accompanied by chlorosis [103]. During the initial colonisation and sporulation phase, these fungi rarely accumulate dark pigments; the conidia, hyphae and fungal projections are typically colourless or light and, due to their texture and density, reflect nearly all incident light without pigment absorption. The absence of pigment synthesis may represent a metabolic-energy saving strategy combined with the fact that the disease develops in lower plant parts, which are shielded from sunlight and therefore do not require UV-protection mechanisms.

Another case is ear blight caused by fusarium species, which is characterised by bleached spikelets and pale-pink mycelial coatings. The mycelial colour may result from polyketide pigments—as seen in *Fusarium fujikuroi* (aurofusarin, bikaverin, rubrofusarin, neurosporaxanthin) [104] and *F. graminearum* (aurofusarin, rubrofusarin) [105]. While this colouring yields somewhat lower reflectance than powdery mildew, it still exceeds that of healthy tissue or dark-pigmented diseases.

Lastly, leaf spot disease (caused by *P. tritici-repentis*) manifests as yellow and brown spots on leaves, stems and grain. In its early phase the yellow spots show up on hyperspectral imagery as red or orange patches. As the disease progresses, brown spots with yellow halos appear, which register as yellow-green patches surrounded by red areas on hyperspectral images. *P. tritici-repentis* produces host-selective toxins (Ptr ToxA, Ptr ToxB, Ptr ToxC) which destroy mesophyll cells and chloroplasts in the infected zone. Loss of chlorophyll causes green to fade, and chlorosis is followed by metabolite accumulation, necrosis and tissue darkening. This mechanism is also observed in other plant diseases, where tissue discolouration or bleaching takes place during the early stages of infection. Overall, leaf blotch diseases are characterised by a prolonged asymptomatic phase during which the pathogen colonises the leaf apoplast, followed by a rapid shift to tissue necrosis and the development of dark-brown pycnidia [106]. This latent stage enables the pathogen to proliferate unobtrusively and evade early host recognition. However, hyperspectral imaging can detect the disease by revealing distinct spectral variations within different regions of the same leaf.

**Figure A3:**
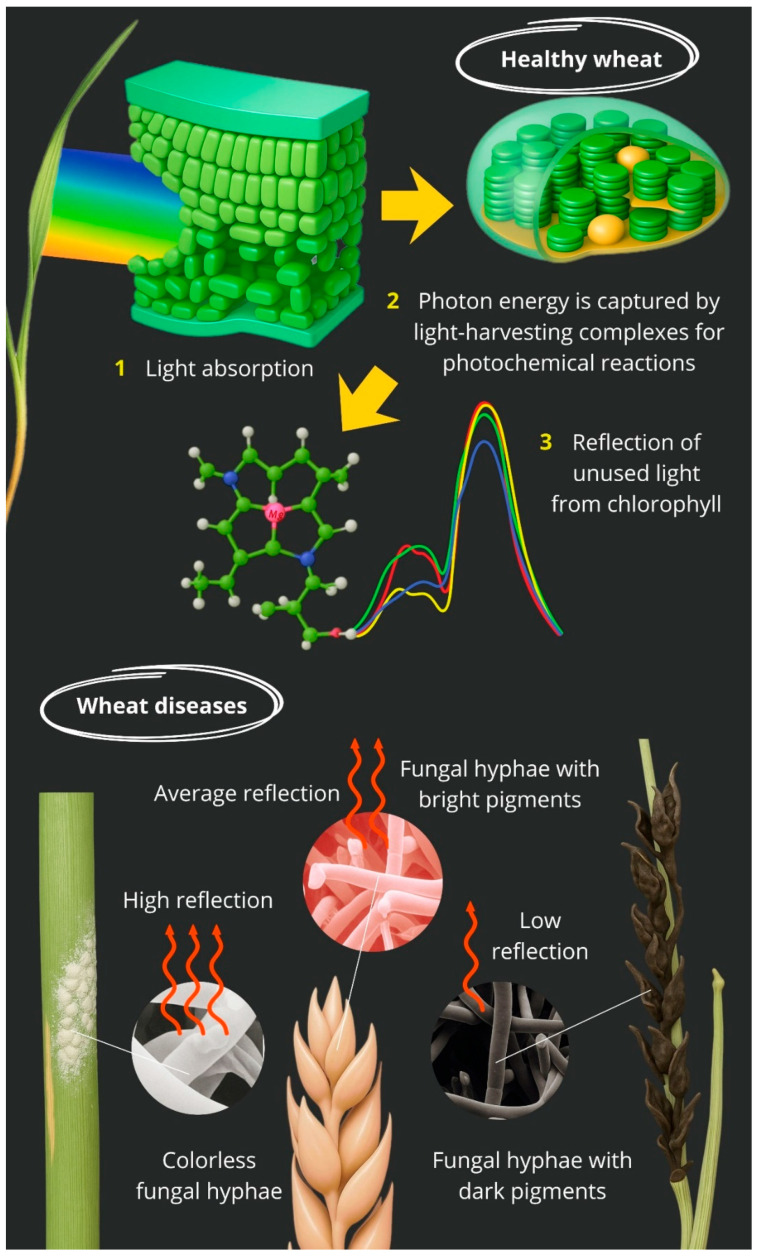
Reflectance changes associated with altered pigment profiles in healthy and diseased plant tissue (one arrow—low reflection; two arrows—average reflection; three arrows—high reflection).

Figure A3 illustrates differences in reflectance behaviour of various pigments between healthy and diseased plant tissue. In healthy plants, tissue reflectance is generally uniform, with variations mainly occurring between different plant organs. This uniformity is a key indicator that distinguishes healthy plants from infected ones, where reflectance becomes patchy due to partially or fully diseased areas. Against the backdrop of healthy tissue, disease symptoms appear as fragmented zones—some highly reflective due to light pigments, others with almost no reflectance due to dark pigmentation.

**Figure A4:**
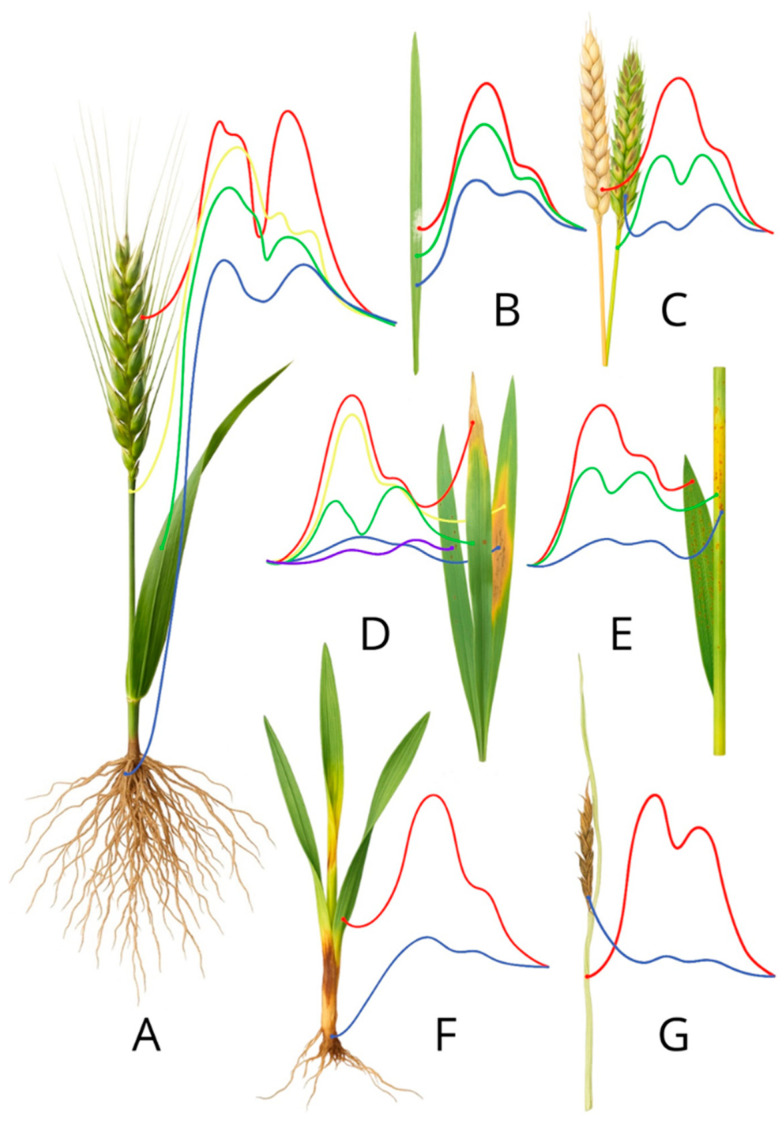
Spectral reflectance patterns of wheat structures affected by different pathogens ((**A**)—heathy; (**B**)—*Blumeria graminis*; (**C**)—*Fusarium graminearum, Parastagonospora nodorum*; (**D**)—*Zymoseptoria tritici, Pyrenophora tritici-repentis, Cochliobolus sativus*; (**E**)—*Puccinia graminis, Puccinia triticina*; (**F**)—*Fusarium* spp., *Bipolaris sorokiniana*; (**G**)—*Ustilago tritici; spectra: red*—*high reflectance, yellow and green*—*medium, blue*—*low*).

The patterns of light reflectance from wheat affected by various diseases are illustrated in Figure A4.

**Figure A5:**
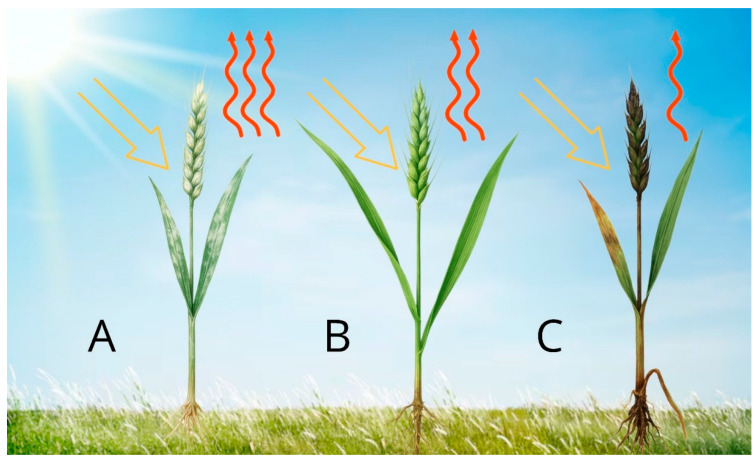
Differences in reflectance intensity by type of pathology ((**A**)—colourless or white-coated diseases; (**B**)—healthy plant; (**C**)—diseases with bright or dark pigments; one arrow—low reflection; two arrows—average reflection; three arrows—high reflection).

Each type of plant disease produces a distinct spectral profile, which largely depends on the biological structure of the pathogen. Diseases with little or no pigmentation, or those featuring white fungal growth, reflect most of the incident light. Diseases involving bright pigments absorb only a small portion of light and reflect the rest strongly. Green pigments like chlorophyll partially absorb light for photosynthesis and reflect the remainder. In contrast, dark pigments such as melanin absorb almost all light, resulting in minimal reflection. Figure A5 clearly illustrates these relationships, showing how the pigment profile of plant tissues influences their spectral response.

**Figure A6:**
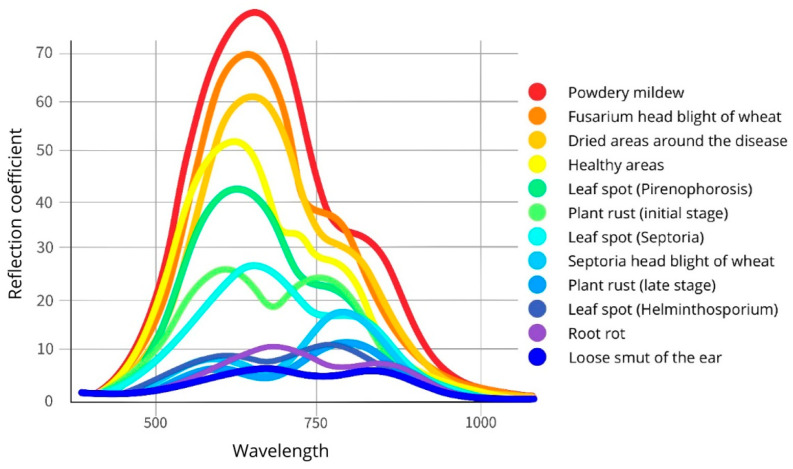
Comparative spectral reflectance profiles of plant samples affected by different types of diseases (*x*-axis—wavelength (nm), *y*-axis—reflectance coefficient (%).

This figure presents spectral reflectance curves across a range of wheat conditions (Figure A6). Diseases characterised by white fungal growth show the highest reflectance. Fusarium head blight and desiccated tissues exhibit slightly lower reflectance than powdery mildew but still higher than that of healthy tissues. Healthy tissues themselves display elevated reflectance in the visible and near-infrared regions, owing to their intact cellular structure and presence of chlorophyll. Leaf spot diseases (e.g., tan spot, septoria tritici blotch, spot blotch) show varying curve profiles depending on the severity and stage of infection. Rust diseases also exhibit a wide spectrum of reflectance behaviour as they progress. The lowest reflectance is observed in root rot and loose smut due to the presence of dark pigments and extensive tissue degradation—factors that increase light absorption and reduce reflectivity.

### 4.3. Spectral Characteristics and Disease Classification

The success of classification models depended less on training dataset size and more on spectral signature contrast, the extent of biophysical tissue changes, and the model’s ability to handle overlapping spectral regions. Misclassifications most often occur when diseases share similar visual symptoms or partially overlapping spectral features. Among the tested algorithms, Random Forest emerged as the most effective for this task. Plant diseases cause complex, nonlinear changes in tissue pigmentation and structure, which are reflected in the spectral data in ways that simple linear models often cannot capture. Random Forest excels at detecting these nonlinear relationships, handling high-dimensional and heterogeneous datasets more accurately than traditional classifiers. Previous studies have shown the successful use of Random Forest in classifying diseases such as wheat and rye rust [107], tomato early blight (*Alternaria solani*) [108], and grapevine powdery mildew [109]. Our study builds on previous efforts by providing a comparative evaluation of classification algorithms for wheat disease detection using hyperspectral imaging. It examines a wide range of pathogens across different stages of infection, providing a comprehensive and representative dataset for assessing the true potential of hyperspectral imaging in plant disease diagnostics.

## 5. Conclusions

In this study, spectral profiles of various wheat diseases were identified and compared with the spectral signatures of healthy wheat samples. The shape and intensity of reflectance curves varied depending on disease type, its developmental stage, and the affected plant organ. Healthy plant tissues exhibited different levels of light reflectance. Despite these differences, overall reflectance variation between plant parts remained relatively small. In hyperspectral imagery, these tissues generally appeared in similar tones without distinct patches or anomalies. Their spectral curves followed consistent shapes without sharp fluctuations.

Diseases producing light-coloured symptoms (powdery mildew or fusarium head blight) exhibited higher reflectance than healthy tissue due to the near-total reflection of light from infected areas. These infections appeared as bright red or orange patches, standing out against the surrounding plant tissue. Their spectral curves contrast sharply with healthy areas because of differences in reflectance between the infection site, the surrounding dried tissues, and unaffected parts of the plant.

Early-stage symptoms (rust, blotch, or septoriosis) manifested as yellow or blue patches on hyperspectral images, typically surrounding red or blue dots that indicate the disease’s focal point. In these cases, plant spectra varied widely: chlorotic areas may display curves with moderate reflectance compared to the “bright” diseases and healthy tissue and higher reflectance compared to “dark” diseases and healthy parts.

Diseases characterised by dark pigmentation resulted from melanin and other compounds production. These symptoms often appear as blue patches or spots on various plant parts. For example, roots with root rot appeared blue; spikes with loose smut showed characteristic patterns; and in spot blotch, numerous blue dots are seen across the plant. The spectral curves of diseased tissues differ significantly from each other, often showing low reflectance values. Moreover, the shapes of these curves vary depending on the specific disease and affected tissue.

Reflectance variability among fungal infections was primarily driven by pathogen-produced pigments. In diseases like root rot and loose smut, melanin production is linked to survival strategies—such as spore dispersal and protection against UV radiation or desiccation. In contrast, the absence of pigment synthesis in some pathogens resulted from their localisation in lower plant parts, which are shaded from sunlight and therefore don’t require such protective responses, allowing pathogens to conserve metabolic energy.

Based on the patterns observed in the spectral characteristics of wheat diseases, a classification model was developed. Among the tested methods, the Random Forest algorithm proved to be the most effective for automated identification of wheat phytopathogens using hyperspectral data. It outperformed other approaches due to its ability to handle high-dimensional, non-linear data, its robustness to noise and class imbalance, and its strong generalisation performance. The resulting model demonstrated high sensitivity to different disease types, resilience to dataset imbalance, and strong precision and recall metrics, achieving an overall classification accuracy of 94%. These findings contribute to the advancement of automated crop health monitoring and underscore the potential of hyperspectral imaging, combined with machine learning algorithms, for early diagnosis and accurate differentiation of grain crop diseases.

This study represents the first comprehensive comparison of spectral profiles of healthy and diseased wheat tissues, taking into account the diversity of diseases, their optical properties, and the biochemical nature of the pigments produced by pathogens. Unlike time-consuming traditional methods that often miss early-stage detection, the hyperspectral approach provides objective, highly accurate, and rapid identification of phytopathogens. This research expands the range of classifiable pathogens and could be integrated into precision agriculture systems that help save resources through localised treatment of affected areas.

## Figures and Tables

**Table 1:** Spectral characteristics of spring wheat samples (healthy and infected by various pathogens).

No.	Healthy/ Disease	Sample	Body Part	Wavelength (nm)	Reflection Coefficient (%)
1	Healthy plant	Healthy stem with a leaf and a root	General	550–800	15–17.5
			Stem	500–780	50–60
			Leaf	500–780	10–25
			Root	500–780	20–40
		Healthy ear with a stem	General	550–800	17–18
			Ear	500–780	30–35
			Stem	500–780	10–25
2	Root rot	Fusarium	General of the leaf	550–780	17.5
			General of the stem	550–780	17.5
			General of the root	550–780	11
			Stem	500–750	40–60
			Leaf	500–750	30–35
			Infected root	500–750	10
		Helminthosporium	General of the leaf	550–780	18
			General of the stem	550–780	16
			General of the root	550–780	13
			Stem	500–750	40–65
			Leaf	500–750	20–35
			Infected root	500–750	10
3	Leaf spot	Tan spot	General	550–780	15–16
			Affected area	550–750	37.5–47
			Healthy area	550–750	22.5–33
		Spot blotch	General	550–780	13–15
			Affected area	550–750	10
			Dried areas	550–750	30–40
			Healthy area	550–750	22.5–25
		Septoria tritici blotch	Affected area	550–750	10–20
			Dried areas around the disease	550–750	30–40
			Healthy area	550–750	22.5–30
4	Plant rust	Ear and stem rust	General	550–780	13–15
			Affected area	550–750	10
			Moderate severity of diseases	550–750	23–40
			Healthy areas	550–750	50–60
		Leaf rust	General		
			Affected areas	550–750	7.5–20
			Moderate severity of diseases	550–750	22.5–45
		Brown stem rust	General		
			Affected areas	550–780	30–50
			Mildly affected areas	550–750	20–30
			Areas at later disease stages	550–750	10
5	Fusarium head blight of wheat		General (ear)	600–780	15–20
			General (stem)	600–780	12.5–15
			Affected areas	550–750	60–70
			Dried areas	600–750	40–50
			Healthy areas	600–750	30
6	Septoria glume blotch		General	550–780	15–17.5
			Affected areas	550–750	10–15
			Dried areas	550–750	50–60
			Healthy areas	550–750	25–35
7	Loose smut	Completely infected	General (ear)	550–780	8
			General (leaf)	550–780	13
			General (stem)	550–780	17.5
			Affected areas	550–750	7
			Dried areas	550–750	15
			Healthy areas	550–750	22.5–37.5
		Partially infected	General (ear)	550–780	11
			General (leaf)	550–780	15
			General (stem)	550–780	17.5
			Affected areas	550–750	7
			Dried areas	550–750	15
			Healthy areas	550–750	22.5–37.5
8	Powdery mildew		Affected areas	550–750	70–80
			Moderate severity of diseases	550–750	50
			Healthy areas	550–750	10–30

**Table 2:** Comparison of classification models based on R^2^Y and Q^2^Y metrics.

Method		R^2^Y	Q^2^Y
Maximum Entropy	SDCA	0.73568	0.68402
Neural Network	AP	0.51738	0.48018
Support Vector Machine	Linear	0.60038	0.55594
Random Forest	Fast Forest	0.74882	0.68527
Soft Independent Modeling of Class Analogy	SIMCA	0.98886	0.99078

**Table 3:** Multidimensional performance evaluation of classification algorithms under variable training data conditions.

Algorithm	Macro Accuracy (R^2^Y)	Log Loss	Log Loss Reduction	Micro Accuracy	Macro Accuracy Test	Cross Validation Macro Accuracy (Q^2^Y)
Maximum Entropy (SDCA)	0.73568	0.73132	0.62742	0.75181	0.70876	0.68402
Neural Network (AP)	0.51738	0.93958	0.51774	0.69626	0.54885	0.48018
Support Vector Machine	0.60038	0.89243	0.53119	0.70813	0.66278	0.55594
Random Forest	0.74882	0.71073	0.63792	0.80964	0.64043	0.68527

**Table 4:** Evaluation metrics for machine learning algorithms.

Metrics	Healthy	*Fusarium* spp.	*Bipolaris sorokiniana*	*Zymoseptoria tritici*	*Pyrenophora tritici-repentis*	*Cochliobolus sativus*	*Puccinia graminis*	***Puccinia triticina***	*Fusarium graminearum*	***Parastagonospora nodorum***	*Ustilago tritici*	*Blumeria graminis*
Random Forest (Fast Forest)												
Precision	98.2	100	93.1	97	85.7	90.5	93	96.4	93.3	95.8	96.7	93.3
Recall	94.7	93.5	96.4	97	89.2	87.4	83.1	92.5	97.6	96.7	87.9	93.3
F1-score	96.4	96.8	94.7	97	87.4	88.9	87.8	93.9	95.4	96.2	92.1	93.3
Maximum Entropy (SDCA)												
Precision	57.8	100.0	76.9	75.6	90.4	91.1	85.0	78.5	86.6	78.8	90.6	100.0
Recall	45.6	65.6	89.3	81.3	88.2	94.3	65.6	48.5	96.4	84.8	87.9	66.7
F1-score	51.0	79.2	82.6	78.3	89.8	92.7	74.0	93.4	91.2	81.7	89.2	80.0
Neural Network (AP)												
Precision	0.0	85.7	64.2	50.6	82.3	72.7	59.4	0.0	78.1	66.3	96.5	0.0
Recall	0.0	37.5	76.8	67.4	64.9	93.0	54.3	0.0	93.0	69.0	63.6	0.0
F1-score	0.0	52.2	69.9	57.8	74.4	81.2	56.7	0.0	85.4	67.6	76.4	0.0
Support Vector Machine												
Precision	0.0	81.2	82.8	66.6	95.0	80.8	76.2	75.0	81.0	71.4	87.5	0.0
Recall	0.0	81.2	85.7	76.8	71.6	92.0	68.5	35.8	59.2	74.9	84.8	0.0
F1-score	0.0	81.2	84.2	71.3	80.9	86.0	72.3	48.5	86.5	73.1	86.2	0.0
SIMCA												
Precision	95.2	100	96.2	86.4	86.1	81.1	43.5	52.5	90.2	43.9	28.5	100
Recall	35.1	90.6	91.1	46.3	83.8	88.5	81.5	47.0	52.3	73.3	84.8	66.7
F1-score	51.3	96.1	93.6	59.5	84.9	84.6	56.7	49.8	66.2	54.9	43.8	80.0

## Data Availability

The data supporting this study’s findings are available on request from the corresponding author.

## Appendix A

Hyperspectral imaging and statistical data processing methodology.

### Appendix A.1. Hyperspectral Imaging

#### Appendix A.1.1. Sample Preparation for Hyperspectral Imaging

Preparation of plant samples infected with different pathogens for hyperspectral imaging followed a standardised procedure. Prior to imaging, panels with a flat matte surface were prepared to minimise reflectance and enable calibration. Plant samples were placed 3–5 cm apart to avoid overlap and ensure proper segmentation during data processing. The distance between the camera and the samples ranged from 30–40 cm, depending on lesion size, lens type, and spatial resolution.

Samples were fixed in their natural orientation—typically with both adaxial and abaxial leaf surfaces exposed—to provide unobstructed access to morphologically significant areas and infected zones. Minimal mechanical contact was maintained to preserve lesion integrity and tissue structure.

Immediately before imaging, white and dark calibration was performed using a Spectralon white reference panel made of fluoropolymer with a high diffuse reflectance coefficient in the visible and near-infrared spectral ranges [68]. This procedure ensured radiometric accuracy and minimised noise in reflectance measurements.

#### Appendix A.1.2. Hyperspectral Image Acquisition and Processing

##### Technical Parameters and Imaging Mode

Hyperspectral imaging was carried out using a FigSpec^®^ FS-13 line-scanning hyperspectral camera (manufacturer: CHNSpec Technology, Hangzhou, China) equipped with a transmission diffraction grating as the spectral dispersive element. The system recorded up to 1200 spectral channels with a spectral resolution (FWHM) of 2.5 nm. The spatial resolution was 1920 pixels per line with a pixel size of 5.86 μm, which is crucial for capturing fine-scale disease lesions in detail. The camera’s CMOS detector provided high sensitivity and low noise—essential for accurate spectral analysis. Imaging was performed at 128 frames per second in full spectral mode, with maximum speed reaching up to 3300 Hz when using region-of-interest (ROI) band selection and allowing the collection of data across the entire VNIR range (400–1000 nm). The camera outputs data over a USB 3.0 interface and uses a C-mount lens attachment. This enabled detailed analysis of the reflectance and absorption properties of various morphological structures and diseased tissue regions [69,70].

##### Image Processing and Preparation for Classification

Hyperspectral image processing and spectral extraction were performed using Breeze (licensed version 2024.2.0) and ENVI (version 5.2) with IDL scripting capabilities, allowing for automated and customisable large-scale data processing. The data were stored as hypercubes—three-dimensional arrays combining two-dimensional spatial information with high spectral resolution.

Preprocessing included radiometric and spectral calibration using “black” and “white” reference images to compute correction coefficients. Spatial masking was then applied to remove background and non-relevant pixels, eliminating unwanted reflections, noise, and glare. Regions of interest (ROIs) were generated using Breeze’s ROI tools or binary thresholding based on spectral homogeneity, employing clustering or morphological filtering algorithms.

Extracted spectral data were subsequently processed through smoothing, Standard Normal Variate (SNV) normalisation, and mean-centring. These steps reduced noise and systematic errors while enhancing the discriminative power of spectral features for classification.

#### Appendix A.1.3. Interactive Spectral Analysis Using PCA and Pixel Explore

Spectral characteristics were analysed using the Pixel Explore tool, which allows users to select sample areas directly on PCA scatter plots. Selected pixels were added to the training dataset with their correct labels. The tool provided Raw Spectrum, Variance Scatter, and Max Variance Image visualisations. In Raw Spectrum plots, each curve represented light reflectance intensity across the 400–1000 nm range for a specific tissue area or lesion. The Variance Scatter plot displayed pixel distribution across the two principal components (t[1] and t[2]), with colour density indicating pixel clustering. The first principal component (t[1]) captured the main spectral differences useful for disease classification, while the second (t[2]) reflected surface texture and subtle lesion morphology. The Max Variance Image, automatically generated in Breeze, projected the hyperspectral cube along the wavelength with maximum variance, facilitating localisation of informative sample regions for further analysis.

#### Appendix A.1.4. Machine Learning Algorithms for Disease Identification and Differentiation

The system was trained to detect, differentiate, and identify the type of pathogen by extracting and creating new feature vectors through combining and optimising spectral characteristics, which were then fed into machine learning classification algorithms. Within the “Model” menu, the Sample statistical model was selected, applying Principal Component Analysis (PCA) and removing background pixels from the images to preserve only the relevant regions of interest (ROI).

For automatic classification and differentiation of disease symptoms in plant samples, various machine learning methods were employed:

- Principal Component Analysis (PCA)—used for dimensionality reduction and to identify major sources of spectral variance.
- Partial Least Squares Discriminant Analysis (PLS-DA)—used to predict categorical variables based on sample spectra.
- Maximum Entropy (SDCA)—a logistic regression model based on maximum entropy, suitable for multiclass problems and large datasets with stochastic optimisation. While fast and scalable, it performs less accurately for nonlinear class boundaries [74].
- Neural Network (AP)—effective for linearly separable data but limited in capturing complex nonlinear patterns, as feature averaging can obscure subtle spectral differences [75].
- Support Vector Machine (SVM)—constructs optimal separating hyperplanes for classification in high-dimensional spaces. Although effective with scaled features, linear versions can struggle with nonlinear class structures [76].
- SIMCA (Soft Independent Modelling of Class Analogy)—builds separate PCA-based models for each class, classifying samples based on their distance to class models. It is useful for small training sets but sensitive to class overlap [77].
- Random Forest (Fast Forest) is a method based on an ensemble of decision trees and represents a faster version of the traditional random forest algorithm, enabling more accurate predictions. It is highly robust to noise and delivers precise results, while also providing feature importance evaluation. This method performs well with linear dependencies and can handle correlated features effectively. In classification tasks, it is capable of combining both spectral data and visual features for improved accuracy [78].

Each model was evaluated using five-fold cross-validation. Manual segmentation tools were employed to mark regions of interest, while the “Grid and Insets” function allowed precise positioning of infected areas and extraction of detailed sub-images for visual inspection. A total of 2184 infected regions were identified and analysed.

The software operated within a spectral range of 391.5–1006.8 nm, covering the visible and near-infrared regions [36]. The spectral range presented actually extends beyond the “effective” working range of the hyperspectral camera sensor, as the sensor retains some sensitivity outside its officially specified physical limits. Therefore, to fully capture the edge values of the spectra within the detector’s capabilities, the true operational range of the device was effectively utilised. Additionally, including the spectral data from the extreme wavelengths—even with lower signal quality—helps improve classification accuracy.

Model training was conducted manually on labeled subsets of pixels, where the class membership (types of spring wheat diseases) was known based on lesion type, morphological characteristics, and localisation. The quality of the classification model was evaluated using cross-validation, relying on accuracy, recall, and F1-score metrics.

### Appendix A.2. Statistical Data Processing

The minimum and maximum reflectance values were calculated using equation:

*R_min_* = *min*(*R*_1_, *R*_2_, …, *R_n_*), (A1)

*R_max_* = *max*(*R*_1_, *R*_2_, …, *R_n_*), (A2)

where *Rₘᵢₙ* is the minimum reflectance value in the sample, *Rₘₐₓ* is the maximum reflectance value, *R*_1_ to *Rₙ* are individual reflectance measurements, and *n* is the total number of measurements.

The mean reflectance value (*μ*), indicating the overall reflectance of the object within the studied spectral range, was calculated using equation:

(A3) $$\mu =\frac{1}{n}\sum_{i=1}^{n}R_{i},$$

where *R_i_* is the reflectance value for the *i*-th measurement, and *n* is the total number of measurements.

The standard deviation (*σ*) represents the dispersion of reflectance values from the mean, providing insight into the variability within the sample. It was calculated using equation:

(A4) $$\sigma =\sqrt{\frac{1}{n}\sum_{i=1}^{n}{{(R}_{i}-\mu )}^{2}},$$

where *μ* is the arithmetic mean of the reflectance coefficient, *Rᵢ* is the reflectance coefficient value for the *i*-th measurement, and *n* is the total number of measurements.

The coefficient of variation (CV) quantifies relative variability, expressing the extent to which the reflectance values deviate from the mean as a percentage. It was calculated using equation:

(A5) $$CV=\frac{\sigma }{\mu }\times 100,$$

where *σ* is the standard deviation, and *μ* is the mean reflectance value.

The reflectance delta (Δ*R*) was calculated using equation to represent the difference between the maximum and minimum reflectance values in a given spectral range or under different conditions:

(A6) $$∆R=R_{max}-R_{min}$$

where *Rₘᵢₙ* is the minimum reflectance coefficient value, and *Rₘₐₓ* is the maximum reflectance coefficient value.

The spectral bandwidth (SB) reflects the width of the spectral range where significant changes in reflectance occur. It helps identify regions with distinct optical properties, such as maximum absorption or reflection. It was calculated using equation:

(A7) $$SB=\lambda_{max}-\lambda_{min},$$

where *λₘᵢₙ* and *λₘₐₓ* are the minimum and maximum wavelengths, respectively.

The rate of change in reflectance makes it possible to track the dynamics of spectral variations. It was calculated using equation:

(A8) $$R=\frac{R_{2}-R_{1}}{\lambda_{2}-\lambda_{1}},$$

The spectral asymmetry (SA) indicates the skewness in the distribution of reflectance values and was calculated using Equation (A8):

(A9) $$SA=\frac{1}{n}\sum_{i=1}^{n}{\left(\frac{R_{i}-\mu }{\sigma }\right)}^{3},$$

where *σ* is the standard deviation, *μ* is the arithmetic mean of the reflectance coefficient, *Rᵢ* is the reflectance coefficient value for the *i*-th measurement, and *n* is the total number of measurements.

Precision represents the proportion of correctly predicted positive cases among all instances the model identified as positive. It is calculated using equation:

(A10) $$Precision=\frac{TP}{TP+FP},$$

where *TP* (true positives) are correctly identified disease cases, and *FP* (false positives) are cases where the model incorrectly indicated the presence of a disease.

Recall measures how well the model detects all actual positive cases and is calculated using equation:

(A11) $$Recall=\frac{TP}{TP+FN},$$

where *FN* (false negatives) are disease cases missed by the model.

F1-score is the harmonic mean of Precision and Recall, providing a balanced metric between the two, and is calculated using equation:

(A12) $$F1=2\times \frac{Precision\times Recall}{Precision+Recall}.$$

## Appendix B

Spectral characteristics of various wheat diseases.

**Table A1:** Spectral characteristics of spring wheat samples (both healthy and infected with various pathogens).

No.	Healthy/ Disease	Sample	Reflection Coefficient (%)								Wavelength (nm)			
			*R_min_*	*R_max_*	*μ*	*σ*	CV	Me	*R*	∆*R*	*λ_min_*	*λ_max_*	SB	~SA
1	Healthy plant	Healthy stem with a leaf and a root	23.75	35.63	29.69	1.51	5.90	29.69	7.65	11.88	513	780	268	0.04
		Healthy ear with a stem	19.00	26.00	22.50	0.89	4.52	22.50	4.51	7.00	517	780	263	0.00
2	Root rot	Fusarium	21.00	25.17	23.08	0.53	1.17	23.08	2.78	4.17	525	765	240	0.00
		Helmintho sporium	19.50	26.17	22.83	0.85	2.16	22.83	4.44	6.67	525	765	240	0.00
3	Leaf spot	Tan spot	25.00	32.00	28.50	0.89	2.83	28.50	5.12	7.00	550	760	210	0.03
		Spot blotch	18.88	22.50	20.69	0.46	1.69	20.69	2.64	3.63	550	758	208	0.04
		Septoria tritici blotch	20.83	30.00	25.42	1.16	5.24	25.42	6.72	9.17	550	750	200	0.02
4	Brown rust	Ear and stem rust	24.00	31.25	27.63	0.92	2.74	27.63	5.30	7.25	550	758	208	0.00
		Leaf rust	15.00	32.50	23.75	2.22	10.01	23.75	12.83	17.50	550	750	200	0.03
		Brown stem rust	20.00	30.00	25.00	1.27	3.81	25.00	7.15	10.00	550	760	210	0.00
5	Fusarium head blight		31.50	37.00	34.25	0.70	2.14	34.25	4.22	5.50	590	762	172	0.03
6	Septoria glume blotch		25.00	31.88	28.44	0.87	3.39	28.44	5.02	6.88	550	758	208	0.04
7	Loose smut	Completely infected	13.83	16.33	15.08	0.32	1.06	15.08	1.83	2.50	550	765	215	0.00
		Partially infected	14.67	17.17	15.92	0.32	1.06	15.92	1.83	2.50	550	765	215	0.00
8	Powdery mildew		43.33	53.33	48.33	1.27	4.80	48.33	7.33	10.00	550	750	200	0.00
